# Effects of a 5-Year Exercise Intervention on White Matter Microstructural Organization in Older Adults. A Generation 100 Substudy

**DOI:** 10.3389/fnagi.2022.859383

**Published:** 2022-06-29

**Authors:** Jasmine Pani, Live Eikenes, Line S. Reitlo, Dorthe Stensvold, Ulrik Wisløff, Asta Kristine Håberg

**Affiliations:** ^1^Department of Neuromedicine and Movement Science, Faculty of Medicine and Health Sciences, Norwegian University of Science and Technology, Trondheim, Norway; ^2^Department of Radiology and Nuclear Medicine, St Olav’s University Hospital, Trondheim, Norway; ^3^Department of Circulation and Medical Imaging, Faculty of Medicine and Health Sciences, Norwegian University of Science and Technology, Trondheim, Norway; ^4^School of Human Movement and Nutrition Sciences, Faculty of Health and Behavioural Sciences, The University of Queensland, Brisbane, QLD, Australia

**Keywords:** cardiorespiratory fitness, neuroimaging, healthy aging, older adults, dose–response relationship

## Abstract

Aerobic fitness and exercise could preserve white matter (WM) integrity in older adults. This study investigated the effect on WM microstructural organization of 5 years of exercise intervention with either supervised moderate-intensity continuous training (MICT), high-intensity interval training (HIIT), or following the national physical activity guidelines. A total of 105 participants (70–77 years at baseline), participating in the randomized controlled trial Generation 100 Study, volunteered to take part in this longitudinal 3T magnetic resonance imaging (MRI) study. The HIIT group (*n* = 33) exercised for four intervals of 4 min at 90% of peak heart rate two times a week, the MICT group (*n* = 24) exercised continuously for 50 min at 70% peak heart rate two times a week, and the control group (*n* = 48) followed the national guidelines of ≥30 min of physical activity almost every day. At baseline and at 1-, 3-, and 5-year follow-ups, diffusion tensor imaging (DTI) scans were performed, cardiorespiratory fitness (CRF) was measured as peak oxygen uptake (VO_2peak_) with ergospirometry, and information on exercise habits was collected. There was no group*time or group effect on any of the DTI indices at any time point during the intervention. Across all groups, CRF was positively associated with fractional anisotropy (FA) and axial diffusivity (AxD) at the follow-ups, and the effect became smaller with time. Exercise intensity was associated with mean diffusivity (MD)/FA, with the greatest effect at 1-year and no effect at 5-year follow-up. There was an association between exercise duration and FA and radial diffusivity (RD) only after 1 year. Despite the lack of group*time interaction or group effect, both higher CRF and exercise intensity was associated with better WM microstructural organization throughout the intervention, but the effect became attenuated over time. Different aspects of exercising affected the WM metrics and WM tracts differently with the greatest and most overlapping effects in the corpus callosum. The current study indicates not only that high CRF and exercise intensity are associated with WM microstructural organization in aging but also that exercise’s positive effects on WM may decline with increasing age.

## Introduction

Promoting healthy brain aging to reduce the risks of dependency and age-related neurological diseases, e.g., Alzheimer’s and related dementias, has become increasingly popular (Livingston et al., 2020). In typical aging, the organization of the brain’s white matter (WM) decreases as both the myelin sheath and axons undergo changes, e.g., dys- and demyelination and axonal loss (Gunning-Dixon et al., 2009; Liu et al., 2017). Decreased WM microstructural organization leads to disrupted signal conduction and is associated with an increased risk of neurodegenerative diseases, changes in cognition, impaired mobility, and poor psychological health (Gunning-Dixon et al., 2009; Debette and Markus, 2010; Madden et al., 2012; Filley and Fields, 2016; Rosso et al., 2017; Fan et al., 2019).

Both physical activity and exercise have been linked to healthy brain aging. According to the cardiorespiratory fitness (CRF) hypothesis, high CRF, a measure of how well the respiratory, circulatory, and muscular systems take up and distribute oxygen during sustained exercise, is considered a central mechanism through which physical activity and exercise support healthy brain aging (Voss, 2016; Voss et al., 2016; Barnes and Corkery, 2018). The exact physiological and molecular underpinning(s) remain(s) to be established but include improved vascular health with increased cerebral blood flow and angiogenesis, as well as an increase in beneficial humoral factors such as brain-derived neurotrophic factor, insulin-like growth factor-1, and decreased inflammation (Cotman et al., 2007; Erickson et al., 2013; Gregory et al., 2013; Voss et al., 2013b,^2016^; Bliss et al., 2021).

Several studies have shown a positive association between CRF and gray matter (GM) volumetric measures from magnetic resonance imaging (MRI) scans (Szabo et al., 2011; Hayes et al., 2013; Raichlen et al., 2019; Pani et al., 2021). Given that the cerebral blood flow is lower in WM than in GM and that WM is largely supplied by end arteries and is prone to small vessel disease in aging (Helenius et al., 2003; Joutel and Chabriat, 2017), CRF could potentially be particularly important for WM health in aging, especially the superior and anterior WM regions, which are shown to be more susceptible to the aging process (Sexton et al., 2014; Bender et al., 2016), could benefit from exercise. Since high-intensity exercise induces higher CRF (Weston et al., 2014), more health-related benefits (Swain and Franklin, 2006), and a lower mortality rate (Wisløff et al., 2006) than moderate-intensity training, we predicted that WM in the HIIT group would benefit most from the intervention. Assessing the effects of different exercise intensity doses in older adults is therefore relevant since surprisingly little is known about the association between WM microstructural organization and CRF.

A few cross-sectional studies report positive relationships between WM fractional anisotropy (FA) and/or mean diffusivity (MD), and measures of CRF (Johnson et al., 2012; Gons et al., 2013; Tian et al., 2014a,b). FA is a measure of WM microstructural organization with higher values reflecting more densely packed and myelinated axons (Seehaus et al., 2015; Friedrich et al., 2020), while MD is considered to indicate the degree of packing or space between cell membranes (Seehaus et al., 2015). There are also studies that do not find associations between CRF and FA and/or MD (Gons et al., 2013; Burzynska et al., 2014). Even a negative association between FA and CRF has been reported (Oberlin et al., 2016). Randomized controlled trials (RCT) and intervention studies conducted during the past decade examining the effects of physical activity and/or exercise on WM microstructural organization are few. One of these studies demonstrated a positive effect on FA, MD, and radial diffusivity (RD) in the fornix of a 6-month dance intervention in healthy older adults (Burzynska et al., 2017), while the FINGER study demonstrated a negative effect of a 2-year multimodal intervention study in older adults at risk for dementia with greater decline in FA in the intervention group than the control group (Stephen et al., 2020). The only systematic review on the effect of exercise on DTI metrics focuses on the corpus callosum and shows greater FA and lower MD mainly located in the mid-anterior region (Loprinzi et al., 2020). However, the vast majority of the intervention or RCT studies spanning from 12 weeks to 24 months did not uncover any group or group*time effects on WM FA/MD in healthy and/or mild cognitive impaired seniors (Voss et al., 2013a; Fissler et al., 2017; Clark et al., 2019; Sejnoha Minsterova et al., 2020; Sexton et al., 2020; Venkatraman et al., 2020). Despite the lack of a group effect of the exercise intervention on WM microstructural organization in late middle-aged-older adults, many of the above studies report associations with different measures of CRF and FA/MD (Voss et al., 2013a; Burzynska et al., 2014; Fissler et al., 2017; Sejnoha Minsterova et al., 2020; Tarumi et al., 2020a). Nevertheless, it remains unclear if there is an optimal exercise type, intensity, or duration which could attenuate age-related WM microstructural organization loss in older adults.

This study was conducted in a subsample of 105 participants from the RCT Generation 100 Study who volunteered for brain MRI before randomization into the intervention groups. The Generation 100 Study investigated the effects of 5 years of supervised exercise as either high-intensity interval training (HIIT), moderate-intensity continuous training (MICT), or following the national physical activity guidelines as the control condition on overall mortality and morbidity in older adults (Stensvold et al., 2015). The Generation 100 Study reported lower mortality trends, significantly higher CRF, and better quality of life in the HIIT compared to the MICT and control groups (Stensvold et al., 2020). The main aim of the current study was to assess the presence of a group*time interaction and group effects and to investigate the relationship between CRF, exercise intensity, and duration on WM FA and MD. Based on the CRF hypothesis and the predominant finding of a positive association between CRF and FA, we expected higher FA and lower MD in the HIIT and MICT groups compared to the control group after 1-, 3-, and 5-years of intervention mainly located to superior-anterior WM regions and the corpus callosum. We also expected CRF, exercise intensity, and duration to be positively associated with FA and negatively with MD in the same WM regions. This is the first 5-year exercise intervention study and the first with HIIT intervention examining WM microstructural organization.

## Materials and Methods

### Study Participants and Intervention

The Generation 100 Study is a registered RCT (NCT01666340, ClinicalTrials.gov registry, ethics approval number 2012/849). The Generation 100 Study aimed to investigate the effect on overall morbidity and mortality of 5 years of supervised exercise at two levels of intensity versus a control group that followed the national physical activity guidelines in older adults from the general population (Stensvold et al., 2015). An invitation letter was sent to all adults born between 1936 and 1942, living permanently and independently in Trondheim County. Of those invited and consenting, 1,567 older adults were eligible for inclusion, i.e., did not participate in other exercise interventions and did not have the somatic disease(s) that precluded exercise or dementia. The included participants were asked if they were interested in also taking part in a neuroimaging study (ethics approval number 2012/381B) and those who agreed to participate and were MRI compatible were included (*N* = 105).

The individuals signing up for the brain MRI substudy had slightly higher CRF and higher educational attainment than those declining or not eligible but were otherwise comparable to the participants in the Generation 100 Study in other clinical and demographic variables (Pani et al., 2021). Participants were first stratified by sex and cohabitation into supervised exercise versus physical activity according to the national physical activity guidelines (1:1), and subsequently, the supervised group was divided into two different levels of exercise intensity (2:1:1) according to the RCT protocol (Stensvold et al., 2015). Group allocation of the participants was performed using a web-based solution by the Unit for Applied Clinical Research (Stensvold et al., 2015). The control group was asked to follow the national physical activity guidelines and perform at least 30 min of moderate-intensity physical activity almost every day (Stensvold et al., 2015). Participants in the MICT and HIIT groups performed exercise sessions two times a week, either supervised or on their own after receiving instructions on exercise intensity and duration. The supervised sessions were conducted both indoors and outdoors and comprised walking, spinning, running, and aerobics, for example (Stensvold et al., 2015). The MICT group performed continuous exercise at 70% of peak heart rate (HR) for 50 min, while the HIIT group performed high-intensity exercise at 85–95% of peak HR for four intervals of 4 min interleaved by active breaks lasting for 3 min. All HIIT and MICT participants had to join a mandatory spinning class led by an exercise physiologist every sixth week wearing an HR monitor to ascertain compliance with their groups’ training intensity.

The Generation 100 Study and the substudy complied with the Declaration of Helsinki, and all participants gave their written informed consent before participating in both studies.

### Demographic and Clinical Data

The data collection is described in detail by Stensvold et al. (2015). Demographic and clinical measurements were acquired at baseline, 1-, 3-, and 5-years. Demographic variables included age, sex, level of education (primary school, high school, and university), cohabitation status (yes/no), and current smoker (yes/no). Clinical measurements were height, body weight, fat and muscle mass percentage, body mass index (BMI), and resting HR. Glucose and triglycerides were measured from fasting blood samples. The participants completed the Short-Form health Survey (SF-8) questionnaire (Ware et al., 2001) to assess health-related quality of life and the Norwegian validated version of the Hospital Anxiety and Depression Scale (HADS) (Zigmond and Snaith, 1983) to measure psychological health. The mental health score from the SF-8 and the total score from HADS are reported. In year 5, general cognition was assessed with the Montreal Cognitive Assessment (MoCA) (Nasreddine et al., 2005). The raw scores are reported, and a likely mild cognitive impairment (MCI) diagnosis was determined based on a score of ≤21 for primary, ≤22 for secondary, and ≤24 for high educational attainment for those aged 75–85 years based on Scandinavian cutoffs (Borland et al., 2017).

The testing of CRF, measured objectively as VO_2_ (mL⋅kg^–1^⋅min^–1^), was performed as a graded maximal exercise test on a treadmill or exercise bike. In participants with prior knowledge of cardiovascular diseases, the test was performed with ECG monitoring, and the testing followed the American College of Cardiology/American Heart Association guidelines (Gibbons et al., 1997). The test started with a 10-min warm up at an individually adjusted submaximal level, after which participants were equipped with a mask for measuring oxygen, blood pressure cuff, pulse belt, and HR electrodes. The test then continued with the same speed and inclination from the last part of the warm up, subsequently about every second minute either the inclination was increased by 2% or the speed was increased by 1 km/h. Maximal oxygen uptake (VO_2max_) was achieved when there was a flattening of VO_2_ (defined as less than 2 ml increase in VO_2_ between two 30-s epochs) despite increased workload, combined with a respiratory exchange ratio ≥ 1.05. For those who could not reach VO_2max_, peak oxygen uptake (VO_2peak_) was measured as the average of the three highest consecutive 10-s VO_2_ registrations. Note that VO_2peak_ was calculated only for participants who stopped the test due to exhaustion but not for participants that stopped the test due to pain or lack of motivation. Since there was a small percentage of participants who could not reach VO_2max_ (34%), hereafter we will use the expression VO_2peak_, which is a combination of VO_2max_ and VO_2peak_ dependent on the participants’ performance as per the RCT protocol.

To assess adherence to the prescribed exercise program, participants filled in a validated self-reported physical activity questionnaire (Kurtze et al., 2007; Stensvold et al., 2015). Adherence to the MICT and HIIT interventions was calculated using questions about exercise frequency, intensity, and duration. Minutes per week of exercising were calculated by multiplying frequency and duration, whereas intensity was based on the Borg 6–20 rating of the perceived exertion scale (Borg, 1982). Adherence to the HIIT program was defined as exercising ≥ 30 min per week at ≥ 15 on the Borg scale; MICT participants adhered if they exercised at least ≥ 30 min weekly at 11–14 on the Borg scale; for controls, adherence was set as ≥ 75 min of exercise per week. For each year, percentage adherence to the prescribed program was calculated as the number of adhering participants divided by the total number of participants at the investigated time point, multiplied by 100.

The types of performed activities were taken from the participants’ answers to the question: “How often do you do the following? (1) Walking: (a) as a way of transport, (b) recreational walking, (c) hiking in nature); (2) Cycling; (3) Swimming; (4) Skiing (in winter); (5) Using fitness center; (6) Organized sports; (7) Other activities.” The possible responses, with the associated scores, were: “Never” (0); “Rarely” (0.25); “1–3 times a month” (0.5); “once a week” (1), “2–3 times a week” (2.5); “4–6 times a week” (5); and “Daily” (7).”

### Magnetic Resonance Imaging Acquisition

All participants underwent an identical standardized MRI protocol acquired on the same 3T Magnetom Skyra (Siemens AG, Erlangen, Germany) with a 32-channel head coil. In this study the DTI scans (*b* = 0 and *b* = 1,000 s/mm^2^; TR = 8,300; TE = 89; FOV = 240 × 240; slice thickness = 2 mm; gap = 0 mm; matrix size = 256 × 256) were used. The DTI sequence was a single-shot balanced-echo EPI sequence acquired in 30 non-collinear directions. A total of sixty transversal slices with no gaps were acquired, giving full brain coverage. Then, five images without diffusion weighting (b0) were acquired to increase the signal-to-noise ratio. To correct for image distortion, two additional b0 images were acquired with opposite phase encoding polarity (b0PA and b0AP) (Holland et al., 2010). Additionally, high resolution 3D T1-weighted MPRAGE (TR = 1,900; TE = 3.16; FOV = 256 × 256; slice thickness = 1 mm; gap = 0 mm) and 3D T2-weighted (TR = 3,200; TE = 412; FOV = 250 × 250; slice thickness = 1 mm; gap = 0 mm) scans were acquired and used to estimate intracranial volume (ICV) with the automatic reverse brain mask (Hansen T. I. et al., 2015) method on SPM8 software package (Wellcome Department of Imaging Neuroscience, London, United Kingdom)^1^.

### Diffusion Image Preprocessing

Diffusion tensor imaging analysis was performed with the FMRIB software library (FSL, Oxford Centre for Functional MRI of the Brain, United Kingdom^2^). Non-brain tissue was removed with the brain extraction tool (BET, FSL). Artifacts due to eddy currents and movements were corrected with eddy (FSL), which simultaneously models the effects of diffusion eddy currents and movements on the image. Additional correction of the susceptibility-induced off-resonance field artifacts was performed by topup (FSL), a tool for estimating and correcting susceptibility-induced distortions (Andersson et al., 2003). Finally, DTIFIT was used to fit a diffusion tensor model to the eddy corrected diffusion data for all individuals, and FA and MD maps were computed. For each participant, head movement was calculated as the average of the motion between volumes based on the eddy corrected image.

### Tract-Based Spatial Statistics

**FIGURE 1:**
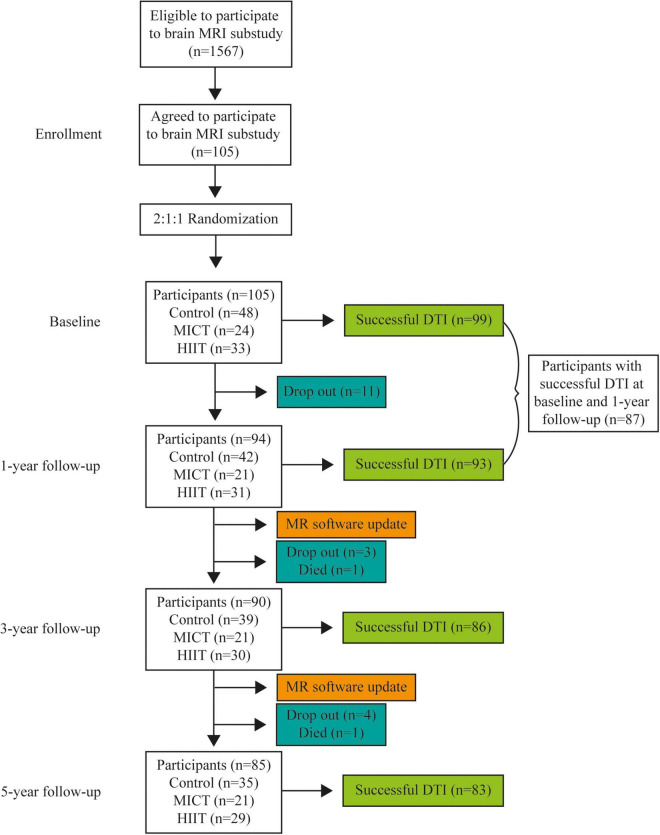
Flowchart of the number of participants (n) in total and each group at every time point and the number of successful DTI scans at each time point. HIIT, high-intensity interval training; MICT, moderate-intensity continuous training.

Using tract-based spatial statistics (TBSS) part of FSL, cross-sectional group comparisons with voxel-wise analysis of whole-brain WM were performed between the MICT, HIIT, and control groups at each follow-up time point during the intervention [1- (*n* = 93), 3- (*n* = 86), and 5-years (*n* = 83), Figure 1] (Smith et al., 2006). Between the 1- and 3-year follow-ups, there was a manufacturer-required MRI software update from Syngo MR D13 to Syngo MR E11. This was followed by a software upgrade to E11C in 2017 before the 5-year MRI collection. These upgrades led to changes in FA and MD values based on quality assessments using healthy human phantoms on site. Similar findings have been published in a DTI phantom study (Timmermans et al., 2019). Since time point and software versions were perfectly confounded in this study, longitudinal analysis was only performed between baseline and 1-year follow-up (*n* = 87), which were obtained with the same software version (Figure 1).

For the cross-sectional analysis, an FA template was made separately for the 1-, 3-, and 5-year follow-up FA data. For the longitudinal analysis from baseline to 1-year, an FA template was made based on all the data from the two-time points. The latter ensured that all images for both time points were in the same image space and orientation. For each of the analyses, all subjects’ FA was aligned to a standard space (FMRIB_58) through a non-linear registration and then affine aligned to MNI space. The registered images were merged and then averaged into a group FA template. The template was thresholded at FA ≥ 0.2 to include WM tracts while excluding peripheral tracts and GM. The FA template was then skeletonized to represent the center of the tracts common to all subjects. Each subject’s aligned FA data was then projected onto this skeleton. For the cross-sectional analysis, the resulting data were fed directly into Randomize for voxelwise cross-subject statistics. For the longitudinal analysis, the skeletonized data was split into a baseline and a 1-year 4D image, and these images were then subtracted to represent change over time.

### Statistical Analysis

#### Group Differences in Demographic, Clinical, and Exercise Characteristics

The two supervised exercise groups and the control group were compared at baseline and 5 years for differences in demographic, clinical characteristics, and head motion using the Chi-squared test, the Kruskal–Wallis test, or a one-way ANOVA as appropriate. Group differences in adherence to the prescribed program at each time point were analyzed with the Kruskal–Wallis test. The same test was used to examine whether there were group differences in exercise frequency, intensity, duration, and performed exercise activity.

#### **Group** D**ifferences in** Diffusion Tensor Imaging M**etrics**

To investigate voxel-wise differences between groups in FA and MD from the TBSS analysis, we used “Randomize,” an FSL tool for non-parametric permutation testing and inference correcting for multiple comparisons. The anatomical locations of significant results on the WM skeleton were identified by superimposing the results on the “JHU ICBM-DTI-81 White Matter Labels Atlas” (Mori et al., 2005).

We investigated group differences longitudinally in participants who had successful DTI scans at baseline and 1-year follow-up to determine if there was a group*time interaction. The longitudinal analysis was corrected for age at baseline, sex, and for exercise intensity and exercise duration measured at baseline. Cross-sectional analyses examined group differences at each time point and were corrected for age at the time of scanning and sex. If there was a significant group or group*time interaction, an additional analysis was performed on axial diffusivity (AxD) and RD.

Supplemental longitudinal and cross-sectional analyses were performed including education and ICV as covariates of no interest (Ho et al., 2011; Takao et al., 2011, 2014).

#### Associations Between Diffusion Tensor Imaging Metrics and Cardiorespiratory Fitness, Exercise Duration, and Intensity Across Groups

To investigate the relationship between WM microstructural organization and different exercise parameters, we evaluated the longitudinal and cross-sectional associations at every follow-up between FA/MD and CRF, exercise intensity, and duration as total weekly minutes exercising, across all groups using Randomize. The analyses were corrected for sex and age at each follow-up. A supplemental analysis was performed with education and ICV as covariates of no interest. If significant associations were present, AxD and RD were examined to investigate the origin of the association.

#### Associations Between Diffusion Tensor Imaging Metrics and Montreal Cognitive Assessment Scores Across Groups

To examine the relationship between WM microstructural organization and MoCA scores, we performed cross-sectional associations between FA/MD and MoCA scores at the 5-year follow-up. The analyses were corrected for sex and age. A supplemental analysis was performed with education and ICV as covariates of no interest. If the association was significant, AxD and RD were also examined.

The mean correlation coefficient “*r*” was calculated for the voxels that showed a significant association between DTI metrics and CRF, exercise intensity, exercise duration, and MoCA, based on the *t*-statistic (*t*) and the degrees of freedom (DoF) with the formula:


$$r=\sqrt{\frac{t^{2}}{t^{2}+\mathrm{DoF}},}$$

where the DoF corresponds to the number of participants at the investigated time point minus 2.

#### Power Calculation and Sample Size

At the time of study design, there was no RCT examining the effect of an exercise intervention on WM microstructural organization. However, based on higher FA in a more physically active group compared to a less active group (Liu et al., 2012) and the positive association between CRF and FA (Marks et al., 2007, 2011; Johnson et al., 2012), we expected to uncover higher FA in groups having fewer than 30 subjects. Furthermore, for a hypothetical 1-year clinical trial, the sample size that is required to show a 50% reduction in the rate of change in FA of the corpus callosum would be 26 participants per arm with a power of 80% and a 2-sided 0.05 alpha. Whereas, for a 2-year clinical trial (with scans at baseline, 1 and 2 years), 7 participants per arm would be needed to show a 50% reduction for a FA yearly decline of the corpus callosum (same alpha/power) (Harrison et al., 2011). Hence, 25 participants in each group in a 5-year intervention study were considered adequate.

## Results

### Group Differences in Demographic, Clinical, and Exercise Characteristics

At baseline, the participants were equally distributed between women (*N* = 52) and men (*N* = 53), had a mean age of 72 years, were predominantly cohabitating (70.2%), and non-smokers (91.3%) with a university education (64.4%). There were no differences between participants in the control, MICT, and HIIT groups on demographic, clinical, psychological, or physical measurements at baseline or at 5-year follow-up (Table 1). No group differences were found in the head motion during DTI scanning between groups. Furthermore, participants that achieved VO_2peak_ and VO_2max_ had similar maximal HR and Borg scores at baseline and each of the follow-ups. This also shows that participants who did not reach VO_2max_ pushed themselves to their limit. Overall, the participants were cognitively intact and had stable cognitive abilities throughout the intervention (Sokołowski et al., 2021). Over the course of the study, 2 participants died and 18 dropped out for unknown reasons. Participants who remained in the study had higher education compared to those who dropped out but were otherwise comparable (Pani et al., 2021).

**TABLE 1:** Demographics, clinical, and cognitive data for the control group, moderate-intensity continuous training (MICT), and high-intensity interval training (HIIT) at baseline and at the end of intervention at 5-year follow-up.

	*Baseline*				*5 years*			
		
	Control (*N* = 48)	MICT (*N* = 24)	HIIT (*N* = 33)	*p*-value	Control (*N* = 35)	MICT (*N* = 21)	HIIT (*N* = 29)	*p*-value
Women^a^ (*%)*	52.1	54.2	42.4	0.61	48.6	52.4	44.8	0.87
**Education^b^**								
*%Primary school*	8.3	12.5	6.2	0.50	2.9	14.3	7.1	0.82
*%High school*	33.3	20.8	21.9		31.4	14.3	17.9	
*%University*	58.3	66.7	71.9		65.7	71.4	75.0	
Cohabitation^a^ (*%Yes)*	68.8	70.8	71.9	0.95	68.8	78.9	72.0	0.74
Current smoker^a^ (*%No)*	89.6	95.7	90.6	0.69	87.5	94.7	92.0	0.67
Age^c^ (years)	71.98 (1.82)	71.75 (1.73)	72.30 (2.11)	0.54	76.6 (1.7)	76.9 (1.8)	77.1 (2.0)	0.58
Height^c^ (cm)	168.95 (9.69)	171.58 (7.45)	170.76 (8.70)	0.44	168.7 (10.5)	170.0 (8.2)	168.6 (7.9)	0.86
Weight^c^ (kg)	74.13 (13.20)	75.71 (9.91)	76.48 (13.56)	0.70	74.0 (15.0)	75.1 (10.8)	74.1 (11.5)	0.96
Fat^c^ (%)	30.25 (7.99)	29.48 (7.81)	28.18 (7.07)	0.49	30.8 (8.3)	31.5 (7.6)	30.0 (5.9)	0.82
Muscle mass^c^ (%)	38.12 (4.78)	38.62 (4.45)	39.23 (4.07)	0.56	37.6 (4.9)	37.3 (4.2)	38.0 (3.4)	0.85
BMI^c^ (kg/m^2^)	25.86 (3.27)	25.86 (3.42)	26.07 (3.28)	0.96	25.9 (3.8)	26.0 (3.6)	26.0 (2.5)	0.99
Resting HR^c^ (beats/min)	63.42 (9.00)	64.96 (8.88)	62.94 (10.43)	0.71	61.6 (8.5)	62.3 (6.2)	60.4 (8.3)	0.72
Glucose^c^ (mmol/L)	5.64 (0.59)	5.44 (0.65)	5.62 (0.77)	0.50	5.3 (0.4)	5.4 (1.1)	5.4 (0.6)	0.71
HDL^c^ (mmol/L)	1.86 (0.56)	1.80 (0.48)	1.89 (0.69)	0.85	1.7 (0.5)	1.7 (0.5)	1.8 (0.5)	0.65
LDL^c^ (mmol/L)	3.62 (0.95)	3.20 (0.66)	3.34 (1.04)	0.15	3.3 (1.1)	2.8 (0.9)	3.2 (0.8)	0.13
Triglycerides^c^ (mmol/L)	1.02 (0.39)	1.00 (0.40)	1.06 (0.61)	0.89	1.0 (0.4)	0.9 (0.4)	1.0 (0.4)	0.30
SF-8 Mental health^b^	55.00 (4.19)	55.39 (4.91)	55.90 (4.30)	0.66	56.0 (5.0)	53.5 (7.5)	57.2 (2.0)	0.68
HADS^c^ (total score)	4.52 (3.50)	4.70 (4.40)	4.53 (3.10)	0.98	6.0 (3.6)	5.9 (5.1)	4.0 (3.3)	0.40
MoCA^c^ (total score)	NA	NA	NA	NA	25.6 (2.7)	26.6 (3.3)	26.4 (2.8)	0.41
**Cardiorespiratory fitness testing**								
VO_2peak_^c^ (mL/min/kg)	29.31 (6.84)	27.21 (5.89)	28.71 (4.92)	0.68	28.48 (6.88)	29.07 (6.82)	29.81 (5.80)	0.93
VO_2max_^c^ (mL/min/kg)	30.84 (6.44)	32.49 (4.36)	30.89 (7.41)	0.74	30.59 (7.88)	28.22 (4.03)	30.78 (6.35)	0.60
HR_max_^c^ (beats/min)	161.60 (13.72)	158.39 (15.60)	159.42 (14.31)	0.63	152.94 (16.46)	153.39 (19.15)	156.75 (14.54)	0.68
Maximal exercise intensity (6–20 Borg scale)	17.27 (1.67)	17.45 (1.47)	17.33 (1.81)	0.91	17.56 (1.25)	17.24 (1.82)	17.57 (1.12)	0.70

*The continuous measures are shown as the mean and standard deviation in the parentheses. Categorical data are reported as percentages.*

*^a^Chi-squared test; ^b^Kruskall–Wallis test; ^c^One-way ANOVA.*

*BMI, body mass index; HADS, hospital anxiety and depression scale; HDL, high-density lipoprotein; HIIT, high-intensity interval training; HR, heart rate; LDL, low-density lipoprotein; MICT, moderate-intensity continuous training; MoCA, Montreal cognitive assessment; NA, not applicable; SF-8, Short-Form health survey questionnaire.*

Exercise intensity during supervised classes showed that participants in the MICT and HIIT groups exercised at a mean of 73 and 88% of peak HR, respectively, corresponding to each group’s prescribed training intensity level. All groups had good adherence to their respective program throughout the 5-year intervention, and there was no difference in percentage adherence between the groups at the follow-up time points (Table 2). Some differences in types of activities performed were present with the HIIT group cycling more in year 1, swimming more in year 3, and using fitness centers more in year 5 compared to the MICT and/or control group (Table 3).

**TABLE 2:** Adherence, exercise frequency, duration, and intensity in the control, MICT, and HIIT groups at each time point.

	Control	MICT	HIIT	Significant difference
**Baseline**				
Exercise frequency (sessions per week)	2.5 (1.2)	3.5 (1.5)	3.2 (1.3)	Control < MICT*
Exercise duration (minutes per session)	43.1 (13.7)	46.6 (11.7)	48.6 (8.4)	–
Min/week exercise	107.8 (58.6)	166.3 (82.1)	160.5 (73.0)	Control < MICT**
Exercise intensity (6–20 Borg scale)	13.2 (2.4)	13.7 (1.5)	14.4 (2.2)	Control < HIIT*
**Year 1**				
Adherence	90.5%	76.2%	74.2%	–
Exercise frequency (sessions per week)	3.0 (1.3)	2.8 (1.3)	3.3 (1.3)	–
Exercise duration (minutes per session)	45.7 (14.4)	46.8 (8.2)	47.9 (9.6)	–
Min/week exercise	140.2 (77.3)	132.3 (75.5)	157.5 (70.9)	–
Exercise intensity (6–20 Borg scale)	13.8 (2.0)	13.6 (0.9)	15.2 (1.5)	Control < HIIT***, MICT < HIIT**
**Year 3**				
Adherence	82.1%	71.4%	86.7%	–
Exercise frequency (sessions per week)	3.0 (1.7)	2.9 (1.2)	3.3 (1.4)	–
Exercise duration (minutes per session)	46.1 (14.0)	49.0 (10.0)	47.5 (12.2)	–
Min/week exercise	146.9 (86.7)	147.8 (53.8)	155.5 (72.5)	–
Exercise intensity (6–20 Borg scale)	13.2 (2.6)	13.4 (0.9)	15.6 (1.3)	Control < HIIT***, MICT < HIIT***
**Year 5**				
Adherence	94.3%	85.7%	79.3%	–
Exercise frequency (sessions per week)	3.3 (1.6)	2.8 (1.3)	3.2 (1.4)	–
Exercise duration (minutes per session)	48.4 (14.5)	50.1 (10.0)	44.4 (13.1)	–
Min/week exercise	168.3 (92.7)	141.1 (75.3)	138.5 (75.9)	–
Exercise intensity (6–20 Borg scale)	13.4 (1.7)	12.5 (2.1)	15.0 (1.4)	Control < HIIT***, MICT < HIIT***

*p ≤ 0.05, **p ≤ 0.01, ***p ≤ 0.001.

*Except for adherence, all values are presented as mean (standard deviation). The post hoc test used is the Dunn’s test.*

*HIIT, high-intensity interval training; MICT, moderate-intensity continuous training; VO_2peak_, peak oxygen uptake.*

**TABLE 3:** Frequency of the performed exercise activity in the control, MICT, and HIIT groups at 1-, 3-, and 5-year follow-ups.

	Control	MICT	HIIT	Significant differences
**Year 1**				
Walking	2.34 (1.20)	2.47 (0.95)	2.43 (1.72)	–
Cycling	0.75 (0.93)	1.03 (2.18)	1.74 (2.09)	Control < HIIT*, MICT < HIIT**
Swimming	0.27 (0.49)	0.21 (0.30)	0.51 (0.76)	–
Skiing (in winter)	0.71 (1.08)	0.71 (1.00)	0.73 (0.92)	–
Fitness center	0.99 (1.19)	0.96 (1.18)	1.47 (1.36)	–
Organized sports	0.15 (0.39)	0.27 (0.49)	0.32 (0.59)	–
Other activities	0.23 (0.66)	0.21 (0.39)	0.53 (0.82)	–
**Year 3**				
Walking	2.26 (1.26)	1.97 (1.36)	2.54 (1.73)	–
Cycling	0.77 (1.16)	1.01 (2.01)	1.54 (1.91)	–
Swimming	0.28 (0.60)	0.09 (0.12)	0.53 (0.66)	Control < HIIT**, MICT < HIIT***
Skiing (in winter)	0.68 (1.10)	0.87 (1.70)	0.72 (0.97)	–
Fitness center	0.87 (1.13)	0.63 (0.83)	1.34 (1.16)	–
Organized sports	0.30 (0.76)	0.27 (0.39)	0.59 (0.99)	–
Other activities	0.50 (0.68)	0.56 (0.65)	0.49 (0.63)	–
**Year 5**				
Walking	2.10 (1.21)	1.81 (1.00)	2.26 (1.63)	–
Cycling	0.78 (1.38)	0.39 (0.78)	1.60 (2.16)	–
Swimming	0.33 (0.92)	0.08 (0.12)	0.43 (0.70)	–
Skiing (in winter)	0.56 (0.92)	0.21 (0.33)	0.49 (0.80)	–
Fitness center	0.91 (1.30)	0.32 (0.64)	1.19 (1.13)	MICT < HIIT**
Organized sports	0.38 (1.10)	0.42 (0.80)	0.51 (0.83)	–
Other activities	0.37 (0.52)	0.61 (0.62)	0.44 (0.40)	–

*p < 0.05, **p ≤ 0.01, ***p ≤ 0.001.

*All values are presented as mean (standard deviation) and represent the self-reported weekly frequency of listed activities. The post hoc test used is the Dunn’s test.*

*HIIT, high-intensity interval training; MICT, moderate-intensity continuous training.*

**FIGURE 2:**
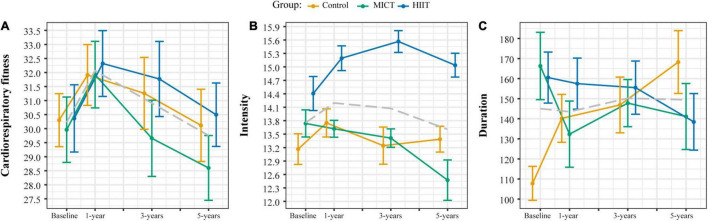
Cardiorespiratory fitness **(A)**, exercise intensity **(B)** measured with the Borg 6–20 scale, and exercise duration **(C)** measured as minutes per week in the control, moderate-intensity continuous training (MICT), and high-intensity interval training (HIIT) groups at each time point. The gray dashed line represents the mean for the whole sample.

Although CRF increased from baseline to 1-year follow-up in all groups, there was no group effect at any time point (Figure 2A). This is in contrast with the main Generation 100 RCT Study, which found higher CRF in the HIIT group compared to the MICT and control groups at each follow-up (Stensvold et al., 2020). At baseline, the control group exercised less frequently and fewer minutes per week than the MICT group and at a lower intensity than the HIIT group (Table 2 and Figure 2B). The control, MICT, and HIIT groups were not statistically different in exercise frequency, exercise duration, and total minutes per week at the 1-, 3-, and 5-year follow-ups (Table 2 and Figures 2B,C). As expected, there was a significant difference in exercise intensity with the HIIT group exercising at a higher intensity than MICT and controls. The control and MICT groups did not differ in terms of intensity (Table 2 and Figure 2B).

### Group Differences in Diffusion Tensor Imaging Metrics

There was no significant group*time interaction from baseline to 1 year on FA/MD in the longitudinal analysis corrected for sex and age, exercise intensity, and duration at baseline.

The cross-sectional analyses revealed no group effect on FA and MD at 1-, 3-, and 5-year follow-ups correcting for sex and age.

There was also no significant group*time interaction or group effect in the supplemental analyses, which included education and ICV as variables of no interest.

Since no group effects were uncovered, the three groups were combined, and associations between the different measures of fitness and exercise on FA and MD were investigated longitudinally as change from baseline to 1 year and cross-sectionally at each follow-up time point across all participants.

### Associations Between Diffusion Tensor Imaging Metrics and Cardiorespiratory Fitness

There was no significant association between change in CRF and change in FA/MD from baseline to 1 year. The association was also not significant when including education and ICV.

**FIGURE 3:**
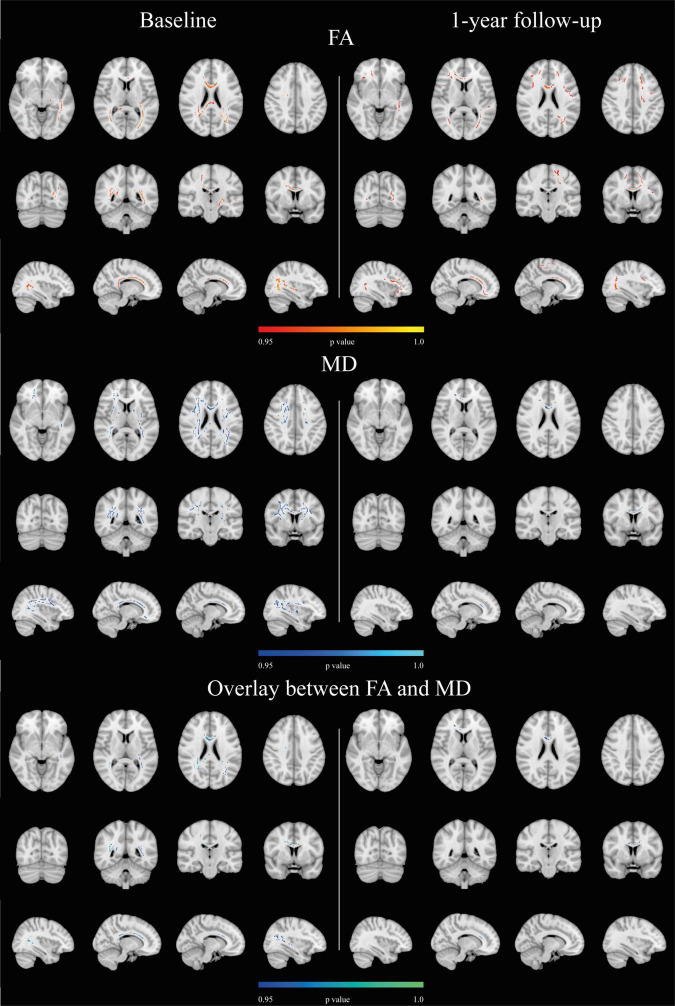
Associations between CRF and DTI metrics. CRF associations with DTI parameters at baseline and at 1-year follow-up (*p* ≤ 0.05, corrected for multiple comparisons, sex, and age). On the bottom row the overlap between FA and MD results. There were no significant results at the 3- or 5-year follow-ups. Results are superimposed on the standard MNI 152 1 mm template on radiological convention. Positive associations are reported in red-yellow, negative relationships in blue-light blue, and the overlap between significant FA and MD results is depicted in blue-green.

Cardiorespiratory fitness was significantly associated with FA and MD at baseline and 1 year (*p* ≤ 0.05, corrected for multiple comparisons, sex, and age) (Figure 3), but not at 3 and 5 years. At baseline, CRF was positively associated with FA in a total of 7,015 voxels (mean *t*-statistic = 2.09, *r* = 0.21) in intrahemispheric, interhemispheric, and projection fiber tracts (Figure 3 and Supplementary Table 1). For MD, negative associations with CRF were present in a total of 13,128 voxels (mean *t*-statistic = 1.97; *r* = 0.2) in intrahemispheric, commissural, and projection fibers (Figure 3 and Supplementary Table 2). The overlap between significant FA and MD accounted for a total of 2,867 voxels (mean *t*-statistic = 2.21; *r* = 0.22) and was mainly located in the posterior regions of the corpus callosum and projection and fronto-occipital association fibers (Figure 3 and Supplementary Table 3). There was a positive association between CRF and AxD, which amounted to 11,332 voxels (mean *t*-statistic = 1.95, *r* = 0.19) mainly located in intrahemispheric, interhemispheric, and projection fiber tracts (Supplementary Table 4). There was no association between CRF and RD at baseline.

After 1-year of intervention, CRF was positively associated with FA in a total of 9,012 voxels (mean *t*-statistic = 1.97; *r* = 0.2) in similar regions as a baseline but more bilaterally distributed (Figure 3 and Supplementary Table 5). For MD, the negative associations comprised a total of 731 voxels (mean *t*-statistic = 2.29; *r* = 0.23) mainly in the corpus callosum (Figure 3 and Supplementary Table 6). The overlap between significant FA and MD was present in 614 voxels in the corpus callosum (mean *t*-statistic = 2; *r* = 0.2) (Figure 3 and Supplementary Table 7). There was a positive association between CRF and AxD, which accounted for a total of 213 voxels (mean *t*-statistic = 3.23; *r* = 0.14) located in the right projection fibers (Supplementary Table 8). There was no association between CRF and RD.

When education and ICV were included in the model, CRF was significantly associated with FA at every follow-up, whereas MD was not. Specifically, CRF and FA were associated in a total of 46,332 voxels at baseline (mean *t*-statistic = 1.86; *r* = 0.18), 44,039 voxels at 1-year (mean *t*-statistic = 1.75; *r* = 0.18), 36,898 voxels at 3-year (mean *t*-statistic = 1.73; *r* = 0.18) and 6,546 voxels at 5-year follow-ups (mean *t*-statistic = 2.01; *r* = 0.22) (Supplementary Tables 9–12). The supplemental analyses on AxD and RD including education and ICV revealed a significant positive association between CRF and AxD at baseline (voxels = 354; mean *t*-statistic = 3.15; *r* = 0.3), 1- (voxels = 750; mean *t*-statistic = 2.98; *r* = 0.3), and 3-year (voxels = 21,450; mean *t*-statistic = 1.77; *r* = 0.19) follow-ups (Supplementary Tables 13–15). No significant associations with RD were present.

### Associations Between Diffusion Tensor Imaging Metrics and Exercise Intensity

There was no significant association between change in exercise intensity and change in FA/MD from baseline to 1-year follow-up. Similarly, no significant association of change was found when education and ICV were included in the model.

**FIGURE 4:**
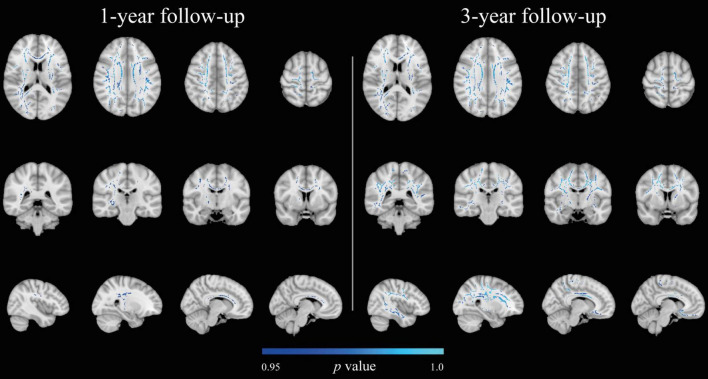
Associations between exercise intensity based on Borg scores and MD at 1- and 3-year follow-ups (*p* ≤ 0.05, corrected for multiple comparisons, sex, and age). There were no significant results at baseline and 5-year follow-up. Results are superimposed on the standard MNI 152 1 mm template on radiological convention. Negative relationships are depicted in blue-light blue.

Fractional anisotropy was not associated with exercise intensity at baseline or the follow-ups. There was a significant negative relationship between MD and exercise intensity at the 1- and 3-year follow-up (*p* ≤ 0.05, corrected for multiple comparisons, sex, and age) (Figure 4), i.e., higher training intensity was associated with lower MD values. At 1-year follow-up, there were a total of 4,972 significant voxels (mean *t*-statistic = 2.11; *r* = 0.21) in intrahemispheric, interhemispheric, and projection fibers associated with training intensity (Supplementary Table 16). At 3-year follow-up, there were 29,842 significant voxels (mean *t*-statistic = 1.89; *r* = 0.2) in the same tracts but extending centrifugally (Supplementary Table 17). No associations were found between MD and the exercise intensity at baseline or 5-year follow-up. No association was also found for the analyses with AxD or RD.

The supplemental analyses with education and ICV in the model revealed a significant positive association between exercise intensity and FA at 1- (voxels = 35,459; mean *t*-statistic = 1.82; *r* = 0.19) and 3-year (voxels = 37,911; mean *t*-statistic = 1.74; *r* = 0.19) follow-ups (Supplementary Tables 18, 19). No significant associations were found between exercise intensity and MD at any follow-up. In the additional analyses with AxD and RD, a positive association between exercise intensity and AxD was found at 1-year follow-up (voxels = 10,059; mean *t*-statistic = 2.13; *r* = 0.22) (Supplementary Table 20). No association was found between exercise intensity and RD.

### Associations Between Diffusion Tensor Imaging Metrics and Exercise Duration

There was no association between change in exercise duration measured as minutes per week and change in FA/MD between baseline and 1-year follow-up. No association of change was found also when including education and ICV in the model.

No association between exercise duration and FA or MD was found at baseline or follow-ups.

In the supplemental analysis with education and ICV as additional covariates, a significant positive association was found between exercise duration and FA (voxels = 906; mean *t*-statistic = 2.47; *r* = 0.25) and a negative association with RD (voxels = 3,622; mean *t*-statistic = 1.96; *r* = 0.2) at 1-year follow-up (Supplementary Tables 21, 22).

### Associations Between Diffusion Tensor Imaging Metrics and

Montreal Cognitive Assessment

At the end of the intervention, there was a significant positive association between MoCA scores and MD, i.e., higher MD values were associated with higher MoCA scores. The negative association was present in a total of 8,802 voxels (mean *t*-statistic = 2.07; *r* = 0.22) (Supplementary Table 23). The additional analysis with AxD and RD revealed that the association was present in the AxD component in 26,943 voxels (mean *t*-statistic = 1.77; *r* = 0.19) (Supplementary Table 24).

When including education and ICV in the model, the association between MoCA and MD disappeared.

## Discussion

In this 5-year exercise intervention in older adults aged 70–77 at baseline, we did not uncover the expected positive effect on WM microstructural organization of supervised exercise intervention with HIIT or MICT compared to following the national physical activity guidelines. We did, however, find some evidence for the predicted positive effect of CRF across all groups on FA and MD. There was also a positive effect of self-reported exercise intensity and duration on DTI indices. Taken together, positive effects of CRF, exercise intensity, and, to some extent, exercise duration were demonstrated on WM microstructural organization, but they were small (*r* = 0.19–0.3). Importantly, all effects did not last for the entire intervention period, despite good adherence to the prescribed training in all groups.

We did not find an effect of the intervention group on WM microstructural organization in any of the analyses, contrary to our hypothesis. However, our finding is consistent with results from the majority of exercise intervention studies lasting from 12 weeks to 24 months performed since the Generation 100 Study started (Voss et al., 2013a; Burzynska et al., 2017; Fissler et al., 2017; Clark et al., 2019; Sejnoha Minsterova et al., 2020; Sexton et al., 2020; Stephen et al., 2020). Only one intervention study has in fact reported a positive group effect of a 6-month dance intervention versus brisk walking, walking, and daily nutritional supplement or a strength, stretching, and balance control group (Burzynska et al., 2017). Since dancing is a type of activity that is not limited to aerobic exercise but also engages the emotional, sensorimotor, visuospatial, and social domains, it could be that this type of activity is more positive for WM microstructural organization. Still, a more recent 6-month dance intervention with similar exercise session duration and frequency could not reproduce the earlier finding (Sejnoha Minsterova et al., 2020). Other interventions such as a 2-year moderate-intensity physical activity compared to usual care in older adults at risk for AD (Venkatraman et al., 2020), a 1-year walking intervention compared to stretching (Voss et al., 2013a), and 6-months of aerobic, strength, coordination, balance, and flexibility training (Fissler et al., 2017) did not uncover group differences, similar to the results in the current study.

The previous intervention studies aimed at increasing aerobic/cardiovascular fitness by implementing moderate-intensity training regimes (Voss et al., 2013a; Sejnoha Minsterova et al., 2020; Venkatraman et al., 2020), i.e., equivalent to our MICT group. The lack of an effect of the MICT intervention is thus in accordance with the findings in these previous shorter-lasting studies, while also demonstrating that a longer duration of MICT did not provide a long-term benefit emerging beyond 2 years. The effect of exercising with HIIT on WM microstructural organization has not been investigated previously. The HIIT group exercised at a higher intensity than the MICT and control groups, and the HIIT intervention had a slightly better effect on CRF in line with previous studies (Ramos et al., 2015; Cao et al., 2019; Sultana et al., 2019; Calverley et al., 2020). However, there was no significant group or group*time interaction effect on FA or MD in the HIIT group, even after the inclusion of CRF, exercise intensity, and duration in the model.

As expected, we found a positive association between FA and CRF in superior and anterior WM regions and the corpus callosum, suggesting that better aerobic fitness can preserve WM microstructural organization in regions prone to age-related changes and in the corpus callosum previously described as sensitive to CRF, exercise, and physical activity (Loprinzi et al., 2020). Our results are consistent with previous studies, which report positive associations between FA and CRF in the corpus callosum (Johnson et al., 2012; Hayes et al., 2015; Oberlin et al., 2016; Tarumi et al., 2020b), the inferior longitudinal fasciculus (Tseng et al., 2013), the internal capsule (Oberlin et al., 2016; Tarumi et al., 2020b), and the superior longitudinal fasciculus (Liu et al., 2012; Tseng et al., 2013; Oberlin et al., 2016; Sejnoha Minsterova et al., 2020; Tarumi et al., 2020b). Additionally, we found positive associations between the thalamic radiation and inferior fronto-occipital fasciculus. Overall, the associations were stronger in the left hemisphere, which has been reported before for exercise effects on brain volumes (Erickson et al., 2011; Firth et al., 2018). Hemispheric differences are present in WM microstructural integrity, and cross-sectional and longitudinal studies report a left-ward asymmetry in aging, with left hemisphere regions showing a larger decrease in FA compared to homologous regions in the right hemisphere (Davatzikos and Resnick, 2002; Bennett et al., 2010). The greater association between CRF and DTI indices in the left hemisphere supports the notion that exercise might slow age-related changes in WM microstructural organization.

After participating in the study for 1 year, FA values in more WM areas in the frontal lobe were associated with CRF compared to baseline. Previous studies report that the anterior WM regions are most vulnerable to the aging process (MacDonald and Pike, 2021). The current results, therefore, suggest that a higher CRF could improve age-related WM microstructural organization in these anterior regions sensitive to aging. Unfortunately, the effect was not present during the entire intervention. In the supplemental analysis including ICV and education as covariates, the positive associations between CRF and FA became more notable and lasted throughout the 5-year intervention although it became attenuated over time as in the main analysis.

In the literature, CRF and MD associations have been less investigated. We found a negative association between CRF and MD in the corpus callosum, the internal and external capsules, and the superior and anterior corona radiata. These results are similar to a recent study on amnestic MCI (Tarumi et al., 2020a), Additionally, we found significant locations in the posterior thalamic radiation. There was a partial overlap of the associations for FA and MD at baseline mainly found in the corpus callosum, right projection, and fronto-occipital association fibers. At 1 year of intervention, the overlap was predominantly present in the corpus callosum. The overlap between MD and FA found in the corpus callosum suggests that CRF was associated with WM microstructural organization in interhemispheric connections. On the other hand, the lack of overlap in frontal regions and fronto-occipital tracts implies that CRF could affect FA and MD somewhat differently and may influence different types of WM tracts differently (i.e., intrahemispheric versus interhemispheric). This was further supported by the supplemental analysis including education and ICV in the model which did not uncover any associations between CRF and MD. Associations between CRF and AxD also waned with time as for FA in the supplemental analysis.

In both the main and supplemental analyses, the positive CRF-FA associations were accompanied by fewer or no MD associations and higher AxD associations, indicating that higher CRF is linked to DTI indices linked to greater axonal packing and myelination. Since the effect on FA and AxD waned over time in both analyses, the positive effect of a higher CRF on WM microstructural organization appears to decrease over time, suggesting that, with increasing age, genetic and/or the cumulative effects of environmental exposure exert a stronger effect on FA than current fitness level.

Exercise intensity was only associated with MD at the 1- and 3-year follow-ups in the main analysis, but only with FA at the 1- and 3-year follow-ups when education and ICV were added to the model, resembling the findings in the CRF analyses. Across all groups, self-reported exercise intensity increased from baseline to 1 year and then declined to the baseline value at 5 years. This could explain why we found an effect of intensity on MD/FA at 1- and 3-year follow-ups but not at baseline and 5-year follow-up. Alternatively, the association between training intensity on WM DTI indices could attenuate over time, like the effect of CRF.

Exercise duration was only associated with DTI indices in the supplemental analysis revealing a significant positive association with FA and negative with RD at the 1-year follow-up. Since the range in exercise duration increase with time, the lack of significant relationships between DTI indices and exercise duration might provide another indication of a lower effect of aspects of exercising with higher age.

Differences across the first year in CRF, exercise intensity, or duration were not associated with any of the DTI indices, thus providing no support to the CRF hypothesis (Voss, 2016).

The cross-sectional analysis, on the other hand, showed that CRF was most strongly associated with the DTI indices followed by exercise intensity, while exercise duration had a very limited or no role in WM microstructural organization. These different aspects of fitness and exercising affected primarily measures related to packing and myelination of axons, and the location of the associations were only partly overlapping. Mode of exercise as reflected by the level of CRF, training intensity, and improvement in strength (Palmer et al., 2013) might therefore exert different effects on WM microstructural organization in different WM tracts with the corpus callosum and the superior longitudinal fasciculus emerging as most consistently positively affected.

### Strengths and Limitations

This study is the first 5-year intervention study including two exercise regiments and comparing them to physical activity according to national guidelines on WM microstructural organization in older adults. The sample was comprised of older adults from the general population of a restricted age range, living independently. Those who agreed to participate in the brain MRI substudy were equally divided between men and women, highly educated, and in good health for the whole intervention. The finding that the participants in the MRI study had higher educational attainment concurs with a previous study in another general population sample (Honningsvåg et al., 2012). That participants who completed the study had higher education compared to those who dropped out might be a limitation, but was also observed in the main study (Viken et al., 2019). Poor recruitment and attrition of participants with a lower education could be a bias in the present study and represents a challenge for future exercise interventions, in particular those including MRI.

Including ICV and education in the statistical models was consistently associated with greater and more widespread associations between CRF, exercise intensity and duration, and the DTI indices, and differences in these variables among participants contributed to differences in WM microstructural organization, which might have obscured effects related to exercising and physical activity. Since including ICV and education in the statistical models did not alter the results of the group comparison, the null finding with regard to training effects appeared consistent.

Unfortunately, the MICT and control groups did not differ in exercise intensity and frequency and these two groups might be too similar to find a statistical difference in WM microstructural organization, which appeared to be associated mainly with CRF and intensity. However, adherence to the prescribed program was high in all groups. In particular, the control group had high adherence to the physical activity national guidelines compared to Norwegian older adults in general (Hansen B. H. et al., 2015). The sample size was comparable to other exercise interventions (Voss et al., 2013a; Burzynska et al., 2017; Sejnoha Minsterova et al., 2020; Venkatraman et al., 2020), and based on the power calculations, it should have been able to reveal group differences.

Exercise in sedentary people leads to higher gains of CRF. On average, the Generation 100 sample was physically active already at baseline, and even though CRF increased, it increased quite similarly in all groups. This could have made uncovering group differences more difficult. Previous studies have shown that although CRF can be increased with exercise at every age, CRF levels undergo a physiological decrease with age which is present also in individuals with high physical activity levels (Aspenes et al., 2011). Therefore, it becomes harder to keep the CRF levels high with increasing age. The finding that the HIIT group maintained a high level of training intensity and still experienced a decline in CRF at the end of the intervention concurs with this.

A limitation was the MRI scanner software update during data collection which interfered with the planned longitudinal analysis across the entire intervention period. Nevertheless, the longitudinal analysis from baseline to 1-year and the cross-sectional analyses at all intervention time points should have been able to uncover group differences. More sophisticated diffusion scan protocols might have been more sensitive to changes in WM related to the interventions. For instance, multishell DTI could have provided better estimates of WM diffusion properties. There are also different approaches than TBSS to analyzing DTI data which might provide information on diffusion in more peripheral WM regions or in specific regions of interest.

Self-report questionnaires on physical activity are commonly used. They are a suitable measure to assess physical activity and are sensitive to cardiovascular health (Wisløff et al., 2006). However, self-report can be imprecise. With increasing age, the perception of effort during exercising has been shown to be assessed as higher than the actual measured effort (Kossi et al., 2021), and there is a tendency of reporting less sedentary time and over reporting the duration of physical activity or exercise (Dyrstad et al., 2014). This would lead to erroneous reporting and hence imprecise estimates of exercise intensity and duration. Smartphones and/or eHealth tools were not common monitoring techniques when the study was initiated in 2012.

Furthermore, not everyone responds equally well to exercise. There is an individual variation in the beneficial effect of exercise with some participants experiencing larger benefits than others (Bouchard and Rankinen, 2001; Ahtiainen et al., 2016; Mattioni Maturana et al., 2021), while some experience deleterious effects (Tuan et al., 2008; Bouchard et al., 2012). Subject-specific exercise prescription that accounts for genetic or environmental contributions or a combination of the two might therefore be advisable to achieve maximal benefits.

## Conclusion

Although we did not uncover a positive effect of partaking in MICT or HIIT on WM microstructural organization compared to following the national physical activity guidelines, we did demonstrate a positive effect of CRF and exercise intensity on WM microstructural organization, which was most notable in the first year of the intervention and which then attenuated toward the end of the intervention period in this 5-year exercise intervention in older adults. The positive associations were located in regions in the WM considered sensitive to aging, suggesting that a high CRF and more intense exercise may positively influence WM aging in adults in their mid-70s, but not at later ages.

## Data Availability Statement

The datasets presented in this article can be accessed by qualified investigators after ethical and scientific review (to ensure the data are being requested for valid scientific research) and must comply with the European Union General Data Protection Regulations (GDPR), Norwegian laws and regulations, and NTNU regulations. The completion of a material transfer agreement (MTA) signed by an institutional official will be required. Requests to access the datasets should be directed to AH, asta.haberg@ntnu.no.

## Ethics Statement

The studies involving human participants were reviewed and approved by Regional Committee for Medical Research Ethics, Central Norway (2012/381B). The patients/participants provided their written informed consent to participate in this study.

## Author Contributions

JP, LR, LE, and AH conceptualized the work, wrote the original draft, and reviewed and edited the work. JP performed the DTI pre-processing. LE overviewed the data QC. JP carried out the statistical analysis and visualization. LR performed the clinical data collection and statistical analyses of the exercise habits. DS and UW were the project administrators of the Generation 100 RCT study, were responsible for acquiring funding and resources, and were responsible for the exercise and clinical data collection. AH was the project administrator of the Generation 100 brain MRI substudy, was responsible for acquiring funding and resources, and was responsible for collecting/organizing the MRI data. All authors contributed to the article and the submitted version.

## Conflict of Interest

The authors declare that the research was conducted in the absence of any commercial or financial relationships that could be construed as a potential conflict of interest.

## Publisher’s Note

All claims expressed in this article are solely those of the authors and do not necessarily represent those of their affiliated organizations, or those of the publisher, the editors and the reviewers. Any product that may be evaluated in this article, or claim that may be made by its manufacturer, is not guaranteed or endorsed by the publisher.

## Footnotes

1
http://www.fil.ion.ucl.ac.uk/spm

2
www.fmrib.ox.ac.uk/fsl
