# Supplementary material for: Effects of a 5-Year Exercise Intervention on White Matter Microstructural Organization in Older Adults. A Generation 100 Substudy
Source: Front Aging Neurosci. 2022 Jun 29;14:859383. doi: 10.3389/fnagi.2022.859383 (PMC9278017; doi:10.3389/fnagi.2022.859383)
Supplement: Supplementary file 1 [file Data_Sheet_1.docx]

**Supplementary Table 1.** Significant clusters of associations between CRF and FA at baseline with sex and age as covariates.

| **Cluster** | **Number of voxels** | **Anatomical regions** | **Peak coordinates (X, Y, Z)** | ***p* value** | **Peak (mean) t-value** |
| --- | --- | --- | --- | --- | --- |
| 5 | 3538 | Genu, body and splenium of corpus callosum, right superior and posterior corona radiata, right anterior and posterior thalamic radiation, right superior and inferior longitudinal fasciculus, right inferior fronto-occipital fasciculus, right tapetum, right corticospinal tract, bilateral cingulum, forceps major and minor | 14, 24, 19 | 0.023 | 4.90 (2.05) |
| 4 | 3347 | Splenium of corpus callosum, left cerebral peduncle, left internal capsule, left posterior corona radiata left anterior and posterior thalamic radiation, left fornix, left superior and inferior longitudinal fasciculus, left inferior fronto-occipital fasciculus, left tapetum, left corticospinal tract, left cingulum, forceps major, left uncinate fasciculus | -36, -51, 6 | 0.018 | 4.56 (2.11) |
| 3 | 64 | Left superior longitudinal fasciculus | -37, -36, 28 | 0.049 | 2.75 (2.17) |
| 2 | 35 | Left superior longitudinal fasciculus | -47, -34, 34 | 0.049 | 3.33 (2.56) |
| 1 | 31 | Left superior longitudinal fasciculus | -43, -44, 27 | 0.049 | 3.55 (2.58) |

*Note.* All significant clusters p ≤ 0.05, corrected for multiple comparisons with a cluster size of at least 30 voxels.

The peak coordinates refer to the maximum intensity voxel in MNI 152 standard space. The *p* value is corrected with the threshold-free cluster enhancement.

**Supplementary Table 2.** Significant clusters of associations between CRF and MD at baseline with sex and age as covariates.

| **Cluster** | **Number of voxels** | **Anatomical regions** | **Peak coordinates (X, Y, Z)** | ***p* value** | **Peak (mean) t-value** |
| --- | --- | --- | --- | --- | --- |
| 5 | 13103 | Genu, body and splenium of corpus callosum, bilateral anterior, posterior and superior corona radiata, bilateral anterior and posterior thalamic radiation, bilateral anterior and posterior internal capsule, bilateral external capsule, bilateral superior and inferior longitudinal fasciculus, bilateral superior and inferior fronto-occipital fasciculus, bilateral corticospinal tract, bilateral cingulum, forceps major and minor, bilateral uncinate fasciculus | 29, -5, 22 | 0.023 | 4.33 (1.97) |

*Note.* All significant clusters p ≤ 0.05, corrected for multiple comparisons with a cluster size of at least 30 voxels.

The peak coordinates refer to the maximum intensity voxel in MNI 152 standard space. The *p* value is corrected with the threshold-free cluster enhancement.

**Supplementary Table 3.** Significant clusters of associations between CRF and the overlap between FA and MD at baseline with sex and age as covariates.

| **Cluster** | **Number of voxels** | **Anatomical regions** | **Peak coordinates (X, Y, Z)** | ***p* value** | **Peak (mean) t-value** |
| --- | --- | --- | --- | --- | --- |
| 35 | 968 | Body and splenium of corpus callosum, right posterior corona radiata, right anterior and posterior thalamic radiation, right superior and inferior longitudinal fasciculus, right inferior fronto-occipital fasciculus, right cingulum, forceps major | 33, -44, 13 | 0.025 | 4.11 (2.18) |
| 34 | 746 | Genu and body of corpus callosum, right superior corona radiata, left cingulum, forceps minor | 19, 4, 32 | 0.026 | 4.90 (2.26) |
| 33 | 628 | Splenium of corpus callosum, left posterior corona radiata, left anterior and posterior thalamic radiation, left superior and inferior longitudinal fasciculus, left inferior fronto-occipital fasciculus, left cingulum, forceps major | -35, -56, 23 | 0.039 | 4.13 (2.22) |
| 32 | 215 | Left anterior thalamic radiation, left superior and inferior longitudinal fasciculus, left inferior fronto-occipital fasciculus, left internal capsule | -29, -23, 1 | 0.042 | 4.33 (2.34) |
| 31 | 84 | Body of corpus callosum, right superior corona radiata, right corticospinal tract | 20, -14, 40 | 0.027 | 3.33 (2.13) |
| 30 | 47 | Left superior longitudinal fasciculus | -38, -42, 23 | 0.041 | 2.75 (2.20) |
| 29 | 33 | Left superior longitudinal fasciculus, left corticospinal tract | -27, -25, 8 | 0.038 | 3.31 (2.20) |
| 28 | 32 | Left superior and inferior longitudinal fasciculus | -35, -66, 25 | 0.044 | 3.27 (2.24) |

*Note.* All significant clusters p ≤ 0.05, corrected for multiple comparisons with a cluster size of at least 30 voxels.

The peak coordinates refer to the maximum intensity voxel in MNI 152 standard space. The *p* value is corrected with the threshold-free cluster enhancement.

**Supplementary Table 4.** Significant clusters of associations between CRF and AD at baseline with sex and age as covariates.

| **Cluster** | **Number of voxels** | **Anatomical regions** | **Peak coordinates (X, Y, Z)** | ***p* value** | **Peak (mean) t-value** |
| --- | --- | --- | --- | --- | --- |
| 24 | 6467 | Splenium of corpus callosum, left anterior corona radiata,  left cerebral peduncle, left anterior and posterior thalamic radiation, left inferior fronto-occipital fasciculus, left superior and inferior longitudinal fasciculus, left corticospinal tract, left cingulum, forceps major and minor, left uncinate fasciculus, internal and external capsule, left fornix | -19, 16, -20 | 0.023 | 5.46 (1.84) |
| 23 | 3385 | Splenium of corpus callosum, right anterior and posterior thalamic radiation, right superior and inferior longitudinal fasciculus, right inferior fronto-occipital fasciculus, right uncinate fasciculus, right cingulum, forceps major, fornix, right internal and external capsule. | 23, -76, 17 | 0.023 | 5.48 (2.03) |
| 22 | 753 | Right anterior thalamic radiation, right corticospinal tract, right internal capsule, forceps minor. | 22, -19, -6 | 0.034 | 4.64 (2.25) |
| 21 | 190 | Left superior and inferior longitudinal fasciculus. | -58, -24, 6 | 0.049 | 3.79 (1.84) |
| 20 | 175 | Left anterior thalamic radiation, left superior longitudinal fasciculus, left inferior fronto-occipital fasciculus, left cingulum, left uncinate fasciculus, forceps minor. | -18, 36, 28 | 0.041 | 5.04 (2.95) |
| 19 | 132 | Left superior and inferior longitudinal fasciculus. | -49, -7, -24 | 0.049 | 3.65 (1.97) |
| 18 | 77 | Right anterior thalamic radiation, right corticospinal tract. | -1, -26, -12 | 0.049 | 3.85 (2.24) |
| 17 | 45 | Left superior longitudinal fasciculus. | -56, -47, 18 | 0.050 | 3.78 (2.15) |
| 16 | 37 | Left superior and inferior longitudinal fasciculus. | -35, -68, 25 | 0.050 | 3.02 (1.97) |

*Note.* All significant clusters p ≤ 0.05, corrected for multiple comparisons with a cluster size of at least 30 voxels.

The peak coordinates refer to the maximum intensity voxel in MNI 152 standard space. The *p* value is corrected with the threshold-free cluster enhancement.

**Supplementary Table 5.** Significant clusters of associations between CRF and FA at 1-year with sex and age as covariates.

| **Cluster** | **Number of voxels** | **Anatomical regions** | **Peak coordinates (X, Y, Z)** | **p value** | **Peak (mean) t-value** |
| --- | --- | --- | --- | --- | --- |
| 16 | 5318 | Genu and body of corpus callosum, right anterior corona radiata, left superior corona radiata, bilateral anterior thalamic radiation, bilateral inferior and superior longitudinal fasciculus, bilateral inferior fronto-occipital fasciculus, left corticospinal tract, bilateral cingulum, forceps minor, bilateral uncinate fasciculus | -16, 30, 36 | 0.031 | 4.91 (1.83) |
| 15 | 1725 | Splenium of corpus callosum, left anterior and posterior thalamic radiation, left superior and inferior longitudinal fasciculus, left inferior fronto-occipital fasciculus, left cingulum, forceps major | -34, -59, 0 | 0.031 | 4.62 (2.20) |
| 14 | 595 | Left superior longitudinal fasciculus | -46, 12, 7 | 0.047 | 4.46 (2.08) |
| 13 | 393 | Right anterior thalamic radiation, left corticospinal tract, left cingulum | -14, -19, 62 | 0.047 | 3.73 (1.83) |
| 12 | 314 | Right anterior and posterior thalamic radiation, right superior and inferior longitudinal fasciculus, right inferior fronto-occipital fasciculus, forceps major | 29, -70, 1 | 0.041 | 4.01 (2.64) |
| 11 | 196 | Right anterior thalamic radiation, right anterior corona radiata, right inferior fronto-occipital fasciculus, right uncinate fasciculus | 28, 30, 10 | 0.049 | 3.07 (1.73) |
| 10 | 172 | Left superior longitudinal fasciculus | -45, -34, 33 | 0.047 | 3.68 (2.33) |
| 9 | 129 | Right superior longitudinal fasciculus | 30, 13, 42 | 0.048 | 4.48 (2.54) |
| 8 | 93 | Right superior longitudinal fasciculus | 34, 34, 25 | 0.049 | 3.19 (1.94) |
| 7 | 87 | Left corticospinal tract | -28, -19, 52 | 0.049 | 2.53 (1.71) |
| 6 | 37 | Right superior longitudinal fasciculus | 35, 2, 29 | 0.049 | 3.42 (2.42) |

*Note.* All significant clusters p ≤ 0.05, corrected for multiple comparisons with a cluster size of at least 30 voxels.

The peak coordinates refer to the maximum intensity voxel in MNI 152 standard space. The *p* value is corrected with the threshold-free cluster enhancement.

**Supplementary Table 6.** Significant clusters of associations between CRF and MD at 1-year with sex and age as covariates.

| **Cluster** | **Number of voxels** | **Anatomical regions** | **Peak coordinates (X, Y, Z)** | ***p* value** | **Peak (mean) t-value** |
| --- | --- | --- | --- | --- | --- |
| 6 | 672 | Genu and body of corpus callosum, right anterior corona radiata, right anterior thalamic radiation, right inferior fronto-occipital fasciculus, left cingulum, forceps minor, right uncinate fasciculus | -3, 15, 20 | 0.046 | 4.93 (2.29) |
| 5 | 62 | Genu and body of corpus callosum, left anterior and superior corona radiata, left cingulum | -18, 15, 28 | 0.05 | 3.27 (2.20) |

*Note.* All significant clusters p ≤ 0.05, corrected for multiple comparisons with a cluster size of at least 30 voxels.

The peak coordinates refer to the maximum intensity voxel in MNI 152 standard space. The *p* value is corrected with the threshold-free cluster enhancement.

**Supplementary Table 7.** Significant clusters of associations between CRF and the overlap between FA and MD at 1-year with sex and age as covariates.

| **Cluster** | **Number of voxels** | **Anatomical regions** | **Peak coordinates (X, Y, Z)** | ***p* value** | **Peak (mean) t-value** |
| --- | --- | --- | --- | --- | --- |
| 11 | 343 | Genu and body of corpus callosum, left cingulum, forceps minor | -3, 15, 20 | 0.046 | 3.77 (2.10) |
| 10 | 179 | Genu of corpus callosum, right anterior corona radiata, right anterior thalamic radiation, right inferior fronto-occipital fasciculus, forceps minor, right uncinate fasciculus | 14, 28, 16 | 0.047 | 3.14 (1.97) |
| 9 | 43 | Body of corpus callosum | -11, -4, 30 | 0.049 | 2.69 (1.83) |

*Note.* All significant clusters p ≤ 0.05, corrected for multiple comparisons with a cluster size of at least 30 voxels.

The peak coordinates refer to the maximum intensity voxel in MNI 152 standard space. The *p* value is corrected with the threshold-free cluster enhancement.

**Supplementary Table 8.** Significant clusters of associations between CRF and AD at 1-year follow-up with sex and age as covariates.

| **Cluster** | **Number of voxels** | **Anatomical regions** | **Peak coordinates (X, Y, Z)** | ***p* value** | **Peak (mean) t-value** |
| --- | --- | --- | --- | --- | --- |
| 1 | 213 | Right anterior thalamic radiation, right corticospinal tract, right cerebral peduncle, right internal capsule | 11, -10, -13 | 0.040 | 4.44 (3.23) |

*Note.* All significant clusters p ≤ 0.05, corrected for multiple comparisons with a cluster size of at least 30 voxels.

The peak coordinates refer to the maximum intensity voxel in MNI 152 standard space. The *p* value is corrected with the threshold-free cluster enhancement.

**Supplementary Table 9.** Significant clusters of associations between CRF and FA at baseline with sex, age education and intracranial volume (ICV) as covariates.

| **Cluster** | **Number of voxels** | **Anatomical regions** | **Peak coordinates (X, Y, Z)** | ***p* value** | **Peak (mean) t-value** |
| --- | --- | --- | --- | --- | --- |
| 2 | 44819 | Genu, body and splenium of corpus callosum, anterior thalamic radiation, anterior, posterior and superior corona radiata, corticospinal tract, superior and inferior longitudinal fasciculus, inferior fronto-occipital fasciculus, uncinate fasciculus, cerebral peduncle, internal and external capsule, cingulum, fornix | -18, 40, 24 | 0.001 | 5.84 (1.86) |
| 1 | 1513 | Left anterior thalamic radiation, left superior longitudinal fasciculus, left inferior fronto-occipital fasciculus, left uncinate fasciculus. | -34, 2, 27 | 0.039 | 4.39 (2.03) |

*Note.* All significant clusters p ≤ 0.05, corrected for multiple comparisons with a cluster size of at least 30 voxels.

The peak coordinates refer to the maximum intensity voxel in MNI 152 standard space. The *p* value is corrected with the threshold-free cluster enhancement.

**Supplementary Table 10.** Significant clusters of associations between CRF and FA at 1-year follow-up with sex, age education and intracranial volume (ICV) as covariates.

| **Cluster** | **Number of voxels** | **Anatomical regions** | **Peak coordinates (X, Y, Z)** | ***p* value** | **Peak (mean) t-value** |
| --- | --- | --- | --- | --- | --- |
| 2 | 43928 | Genu, body and splenium of corpus callosum, anterior and posterior thalamic radiation, anterior, posterior and superior corona radiata, corticospinal tract, superior and inferior longitudinal fasciculus, inferior fronto-occipital fasciculus, uncinate fasciculus, right cerebral peduncle, internal and external capsule, cingulum, fornix, forceps major and minor | 22, -78, 19 | 0.004 | 5.07 (1.75) |
| 1 | 111 | Left cingulum. | -10, -44, 41 | 0.049 | 3.50 (2.10) |

*Note.* All significant clusters p ≤ 0.05, corrected for multiple comparisons with a cluster size of at least 30 voxels.

The peak coordinates refer to the maximum intensity voxel in MNI 152 standard space. The *p* value is corrected with the threshold-free cluster enhancement.

**Supplementary Table 11.** Significant clusters of associations between CRF and FA at 3-year follow-up with sex, age education and intracranial volume (ICV) as covariates.

| **Cluster** | **Number of voxels** | **Anatomical regions** | **Peak coordinates (X, Y, Z)** | ***p* value** | **Peak (mean) t-value** |
| --- | --- | --- | --- | --- | --- |
| 3 | 36866 | Genu, body and splenium of corpus callosum, cerebral peduncle, anterior and posterior thalamic radiation, anterior, posterior and superior corona radiata, corticospinal tract, superior and inferior longitudinal fasciculus, inferior fronto-occipital fasciculus, uncinate fasciculus, cingulum, external and internal capsule, forceps major and minor. | 12, -31, 26 | 0.006 | 4.69 (1.72) |

*Note.* All significant clusters p ≤ 0.05, corrected for multiple comparisons with a cluster size of at least 30 voxels.

The peak coordinates refer to the maximum intensity voxel in MNI 152 standard space. The *p* value is corrected with the threshold-free cluster enhancement.

**Supplementary Table 12.** Significant clusters of associations between CRF and FA at 5-year follow-up with sex, age education and intracranial volume (ICV) as covariates.

| **Cluster** | **Number of voxels** | **Anatomical regions** | **Peak coordinates (X, Y, Z)** | ***p* value** | **Peak (mean) t-value** |
| --- | --- | --- | --- | --- | --- |
| 14 | 2727 | Genu and body of corpus callosum, anterior thalamic radiation, anterior corona radiata, right superior longitudinal fasciculus, right inferior fronto-occipital fasciculus, right uncinate fasciculus, cingulum, | 23, 30, -5 | 0.034 | 3.89 (1.97) |
| 13 | 1310 | Left anterior and posterior thalamic radiation, left superior and inferior longitudinal fasciculus, left inferior fronto-occipital fasciculus, left cingulum, forceps major, left internal capsule | -37, -40, 4 | 0.037 | 4.12 (2.03) |
| 12 | 1163 | Body and splenium of corpus callosum, left anterior and posterior thalamic radiation, left posterior corona radiata, left superior and inferior longitudinal fasciculus, left inferior fronto-occipital fasciculus, left cingulum, forceps major | -16, -42, 10 | 0.045 | 3.49 (1.92) |
| 11 | 598 | Body and splenium of corpus callosum, right anterior and posterior thalamic radiation, right posterior corona radiata, right superior and inferior longitudinal fasciculus, right inferior fronto-occipital fasciculus, right cingulum, forceps major | 16, -43, 21 | 0.044 | 3.91 (2.01) |
| 10 | 309 | Left anterior thalamic radiation, left superior longitudinal fasciculus, left inferior fronto-occipital fasciculus, left cingulum, forceps minor | -17, 49, 15 | 0.047 | 4.20 (2.34) |
| 9 | 185 | Left superior and inferior longitudinal fasciculus | -51, -23, 1 | 0.049 | 3.73 (1.99) |
| 8 | 99 | Right superior longitudinal fasciculus, right corticospinal tract | 30, -27, 39 | 0.049 | 3.82 (2.27) |
| 7 | 52 | Left inferior longitudinal fasciculus | -38, -30, -20 | 0.050 | 2.79 (1.80) |
| 6 | 38 | Left superior and inferior longitudinal fasciculus, left inferior fronto-occipital fasciculus, forceps major | 25, -73, 15 | 0.049 | 2.82 (2.19) |

*Note.* All significant clusters p ≤ 0.05, corrected for multiple comparisons with a cluster size of at least 30 voxels.

The peak coordinates refer to the maximum intensity voxel in MNI 152 standard space. The *p* value is corrected with the threshold-free cluster enhancement.

**Supplementary Table 13.** Significant clusters of associations between CRF and AD at baseline with sex, age education and intracranial volume (ICV) as covariates.

| **Cluster** | **Number of voxels** | **Anatomical regions** | **Peak coordinates (X, Y, Z)** | ***p* value** | **Peak (mean) t-value** |
| --- | --- | --- | --- | --- | --- |
| 5 | 175 | Left anterior and posterior thalamic radiation, left superior and inferior fasciculus, left interior fronto-occipital fasciculus, forceps major | -25, -67, 15 | 0.041 | 4.63 (2.86) |
| 4 | 125 | Right anterior thalamic radiation, right corticospinal tract, right cerebral peduncle | 11, -4, -5 | 0.045 | 4.26 (3.15) |
| 3 | 40 | Right inferior longitudinal and fronto-occipital fasciculus, forceps major | 23, -76, 17 | 0.044 | 5.37 (4.20) |

*Note.* All significant clusters p ≤ 0.05, corrected for multiple comparisons with a cluster size of at least 30 voxels.

The peak coordinates refer to the maximum intensity voxel in MNI 152 standard space. The *p* value is corrected with the threshold-free cluster enhancement.

**Supplementary Table 14.** Significant clusters of associations between CRF and AD at 1-year follow-up with sex, age education and intracranial volume (ICV) as covariates.

| **Cluster** | **Number of voxels** | **Anatomical regions** | **Peak coordinates (X, Y, Z)** | ***p* value** | **Peak (mean) t-value** |
| --- | --- | --- | --- | --- | --- |
| 4 | 409 | Right anterior thalamic radiation, right corticospinal tract, right cerebral peduncle, right internal capsule. | 11, -10, -13 | 0.032 | 4.33 (2.83) |
| 3 | 174 | Right anterior and posterior thalamic radiation, inferior longitudinal and fronto-occipital fasciculus, forceps major. | 32, -63, 2 | 0.041 | 5.02 (3.02) |
| 2 | 120 | Left anterior and posterior thalamic radiation, left superior and inferior longitudinal fasciculus, left inferior fronto-occipital fasciculus, forceps major. | -34, -61, 2 | 0.043 | 5.04 (3.43) |
| 1 | 47 | Right posterior thalamic radiation, right superior and inferior longitudinal fasciculus, right inferior fronto-occipital fasciculus. | 40, -42, -3 | 0.049 | 4.00 (3.07) |

*Note.* All significant clusters p ≤ 0.05, corrected for multiple comparisons with a cluster size of at least 30 voxels.

The peak coordinates refer to the maximum intensity voxel in MNI 152 standard space. The *p* value is corrected with the threshold-free cluster enhancement.

**Supplementary Table 15.** Significant clusters of associations between CRF and AD at 3-year follow-up with sex, age education and intracranial volume (ICV) as covariates.

| **Cluster** | **Number of voxels** | **Anatomical regions** | **Peak coordinates (X, Y, Z)** | ***p* value** | **Peak (mean) t-value** |
| --- | --- | --- | --- | --- | --- |
| 15 | 17978 | Genu, body and splenium of corpus callosum, cerebral peduncle, anterior and posterior thalamic radiation, anterior, posterior and superior corona radiata, corticospinal tract, superior and inferior longitudinal fasciculus, inferior fronto-occipital fasciculus, uncinate fasciculus, cingulum, external and internal capsule, forceps major and minor, fornix. | 22, -77, 18 | 0.013 | 5.57 (1.74) |
| 14 | 1888 | Splenium of corpus callosum, left anterior and posterior thalamic radiation, left corticospinal tract, left posterior corona radiata, left superior and inferior longitudinal fasciculus, left inferior fronto-occipital fasciculus, uncinate fasciculus, cingulum, left external and internal capsule, fornix | -33, -60, 1 | 0.028 | 5.03 (2.01) |
| 13 | 421 | Right anterior thalamic radiation, right superior and inferior longitudinal fasciculus, right inferior fronto-occipital fasciculus, right cingulum, forceps major | 18, -67, 41 | 0.047 | 3.98 (1.78) |
| 12 | 282 | Left superior longitudinal fasciculus, left corticospinal tract | -32, -14, 38 | 0.048 | 3.15 (1.88) |
| 11 | 274 | Right superior and inferior longitudinal fasciculus, right inferior fronto-occipital fasciculus, right uncinate fasciculus, right cingulum | 43, -9, -26 | 0.047 | 4.48 (1.81) |
| 10 | 156 | Left superior longitudinal fasciculus | -44, -45, 35 | 0.048 | 3.51 (1.83) |
| 9 | 97 | Left anterior thalamic radiation, left corticospinal tract, left cerebral peduncle | -5, -26, -32 | 0.048 | 4.43 (2.04) |
| 8 | 93 | Left superior and inferior longitudinal fasciculus, left inferior fronto-occipital fasciculus. | -35, -35, 24 | 0.049 | 3.16 (1.85) |
| 7 | 76 | Left superior longitudinal fasciculus, left inferior fronto-occipital fasciculus | -32, -34, 37 | 0.050 | 3.40 (1.85) |
| 6 | 65 | Right posterior corona radiata, right cingulum. | 14, -37, 38 | 0.050 | 2.78 (1.91) |
| 5 | 59 | Splenium of corpus callosum, left anterior thalamic radiation, left cingumu, forceps major | -18, -39, 25 | 0.049 | 3.85 (2.03) |
| 4 | 45 | Left superior longitudinal fasciculus | -40, 8, 16 | 0.049 | 3.61 (2.30) |

*Note.* All significant clusters p ≤ 0.05, corrected for multiple comparisons with a cluster size of at least 30 voxels.

The peak coordinates refer to the maximum intensity voxel in MNI 152 standard space. The *p* value is corrected with the threshold-free cluster enhancement.

**Supplementary Table 16.** Significant clusters of associations between exercise intensity and MD at 1-year with sex and age as covariates.

| **Cluster** | **Number of voxels** | **Anatomical regions** | **Peak coordinates (X, Y, Z)** | ***p* value** | **Peak (mean) t-value** |
| --- | --- | --- | --- | --- | --- |
| 1 | 4972 | Genu, body and splenium of corpus callosum, right anterior corona radiata, bilateral superior corona radiata, right posterior corona radiata, bilateral anterior thalamic radiation, right posterior thalamic radiation, bilateral superior longitudinal fasciculus, right inferior longitudinal fasciculus, right inferior fronto-occipital fasciculus, bilateral cingulum, bilateral corticospinal tract, forceps major and minor, right anterior and posterior limb of internal capsule, external capsule | 27, -26, -2 | 0.034 | 4.63 (2.11) |

*Note.* All significant clusters p ≤ 0.05, corrected for multiple comparisons with a cluster size of at least 30 voxels.

The peak coordinates refer to the maximum intensity voxel in MNI 152 standard space. The *p* value is corrected with the threshold-free cluster enhancement.

**Supplementary Table 17.** Significant clusters of associations between exercise intensity and MD at 3-years with sex and age as covariates.

| **Cluster** | **Number of voxels** | **Anatomical regions** | **Peak coordinates (X, Y, Z)** | ***p* value** | **Peak (mean) t-value** |
| --- | --- | --- | --- | --- | --- |
| 1 | 29842 | Genu, body and splenium of corpus callosum, bilateral anterior, posterior and superior corona radiata, right anterior thalamic radiation, bilateral posterior thalamic radiation, bilateral superior longitudinal fasciculus, bilateral inferior longitudinal fasciculus, bilateral inferior fronto-occipital fasciculus, bilateral cingulum, bilateral corticospinal tract, forceps major and minor, right anterior and posterior limb of internal capsule, external capsule, uncinate fasciculus | 35, -13, 35 | 0.01 | 6.35 (1.89) |

*Note.* All significant clusters p ≤ 0.05, corrected for multiple comparisons with a cluster size of at least 30 voxels.

The peak coordinates refer to the maximum intensity voxel in MNI 152 standard space. The *p* value is corrected with the threshold-free cluster enhancement.

**Supplementary Table 18.** Significant clusters of associations between exercise intensity and FA at 1-year follow-up with sex, age education and intracranial volume (ICV) as covariates.

| **Cluster** | **Number of voxels** | **Anatomical regions** | **Peak coordinates (X, Y, Z)** | ***p* value** | **Peak (mean) t-value** |
| --- | --- | --- | --- | --- | --- |
| 5 | 34183 | Genu, body and splenium of corpus callosum, anterior and posterior thalamic radiation, anterior, posterior and superior corona radiata, corticospinal tract, superior and inferior longitudinal fasciculus, inferior fronto-occipital fasciculus, uncinate fasciculus, cerebral peduncle, internal and external capsule, cingulum, fornix, forceps major and minor | 41, -25, -17 | 0.003 | 5.36 (1.82) |
| 4 | 1267 | Body of corpus callosum, anterior and superior corona radiata, left corticospinal tract, left superior longitudinal fasciculus, left inferior fronto-occipital fasciculus, left cingulum, forceps minor | -10, 38, 42 | 0.045 | 4.22 (1.75) |

*Note.* All significant clusters p ≤ 0.05, corrected for multiple comparisons with a cluster size of at least 30 voxels.

The peak coordinates refer to the maximum intensity voxel in MNI 152 standard space. The *p* value is corrected with the threshold-free cluster enhancement.

**Supplementary Table 19.** Significant clusters of associations between exercise intensity and FA at 3-year follow-up with sex, age education and intracranial volume (ICV) as covariates.

| **Cluster** | **Number of voxels** | **Anatomical regions** | **Peak coordinates (X, Y, Z)** | ***p* value** | **Peak (mean) t-value** |
| --- | --- | --- | --- | --- | --- |
| 1 | 37911 | Genu, body and splenium of corpus callosum, anterior and posterior thalamic radiation, anterior, posterior and superior corona radiata, corticospinal tract, superior and inferior longitudinal fasciculus, inferior fronto-occipital fasciculus, uncinate fasciculus, cerebral peduncle, internal and external capsule, cingulum, fornix, forceps major and minor | -8, 6, 26 | 0.015 | 4.75 (1.74) |

*Note.* All significant clusters p ≤ 0.05, corrected for multiple comparisons with a cluster size of at least 30 voxels.

The peak coordinates refer to the maximum intensity voxel in MNI 152 standard space. The *p* value is corrected with the threshold-free cluster enhancement.

**Supplementary Table 20.** Significant clusters of associations between exercise intensity and AD at 1-year follow-up with sex, age education and intracranial volume (ICV) as covariates.

| **Cluster** | **Number of voxels** | **Anatomical regions** | **Peak coordinates (X, Y, Z)** | ***p* value** | **Peak (mean) t-value** |
| --- | --- | --- | --- | --- | --- |
| 21 | 3836 | Splenium of corpus callosum, left anterior and posterior thalamic radiation, left posterior corona radiata, left superior longitudinal fasciculus, left inferior fronto-occipital fasciculus, left uncinate fasciculus, cingulum, forceps major | -21, -81, 14 | 0.024 | 5.17 (1.97) |
| 20 | 2084 | Left anterior thalamic radiation, left anterior corona radiata, left superior longitudinal fasciculus, left inferior fronto-occipital fasciculus, left uncinate, cingulum, forceps minor | -26, 30, -5 | 0.039 | 4.56 (2.12) |
| 19 | 1539 | Splenium of corpus callosum, right anterior and posterior thalamic radiation, right posterior corona radiata, right superior and inferior longitudinal fasciculus, right inferior fronto-occipital fasciculus, right cingulum, forceps major | 30, -69, 3 | 0.022 | 4.94 (2.33) |
| 18 | 655 | Right anterior thalamic radiation, right anterior corona radiata, right superior longitudinal fasciculus, right inferior fronto-occipital fasciculus, right uncinate fasciculus, forceps minor | 31, 22, 17 | 0.039 | 4.58 (2.25) |
| 17 | 560 | Left anterior thalamic radiation, left superior and inferior longitudinal fasciculus, left inferior fronto-occipital fasciculus, left uncinate fasciculus, left cingulum | -47, -8, -11 | 0.046 | 4.32 (1.96) |
| 16 | 377 | Right anterior thalamic radiation, right inferior fronto-occipital fasciculus, right cingulum, forceps minor | 19, 19, 41 | 0.046 | 3.92 (2.19) |
| 15 | 279 | Right anterior corona radiata, right anterior thalamic radiation, right inferior fronto-occipital fasciculus, right uncinate fasciculus, right cingulum, forceps minor | 16, 50, 20 | 0.045 | 4.66 (2.28) |
| 14 | 182 | Genu of corpus callosum, cingulum, forceps minor | -9, 24, -7 | 0.046 | 4.12 (2.21) |
| 13 | 179 | Right anterior thalamic radiation, right inferior fronto-occipital fasciculus, right uncinate fasciculus, forceps minor | 15, 42, -13 | 0.046 | 3.37 (2.37) |
| 12 | 149 | Left anterior thalamic radiation, left anterior corona radiata, left superior longitudinal fasciculus, left inferior fronto-occipicatl fasciculus, left uncinate fasciculus, left external capsule | -27, 15, 7 | 0.045 | 4.76 (2.71) |
| 11 | 124 | Left inferior fronto-occipital fasciculus, left uncinate fasciculus, left external capsule | -22, 13, -13 | 0.045 | 5.21 (2.87) |
| 10 | 31 | Left anterior thalamic radiation, left inferior fronto-occipital fasciculus, left uncinate fasciculus, left cingulum, forceps minor | -13, 59, 4 | 0.049 | 3.70 (2.30) |

*Note.* All significant clusters p ≤ 0.05, corrected for multiple comparisons with a cluster size of at least 30 voxels.

The peak coordinates refer to the maximum intensity voxel in MNI 152 standard space. The *p* value is corrected with the threshold-free cluster enhancement.

**Supplementary Table 21.** Significant clusters of associations between exercise duration and FA at 1-year follow-up with sex, age education and intracranial volume (ICV) as covariates.

| **Cluster** | **Number of voxels** | **Anatomical regions** | **Peak coordinates (X, Y, Z)** | ***p* value** | **Peak (mean) t-value** |
| --- | --- | --- | --- | --- | --- |
| 3 | 594 | Left anterior thalamic radiation, left superior corona radiata, left corticospinal tract, left superior longitudinal fasciculus, internal and external capsule | -32, -1, 21 | 0.045 | 5.28 (2.26) |
| 2 | 282 | Left superior longitudinal fasciculus | -18, 3, 46 | 0.041 | 4.98 (2.85) |

*Note.* All significant clusters p ≤ 0.05, corrected for multiple comparisons with a cluster size of at least 30 voxels.

The peak coordinates refer to the maximum intensity voxel in MNI 152 standard space. The *p* value is corrected with the threshold-free cluster enhancement.

**Supplementary Table 22.** Significant clusters of associations between exercise duration and RD at 1-year follow-up with sex, age education and intracranial volume (ICV) as covariates.

| **Cluster** | **Number of voxels** | **Anatomical regions** | **Peak coordinates (X, Y, Z)** | ***p* value** | **Peak (mean) t-value** |
| --- | --- | --- | --- | --- | --- |
| 4 | 3597 | Genu of corpus callosum, left anterior thalamic radiation, left anterior and superior corona radiata, left corticospinal tract, left superior longitudinal fasciculus, left uncinate fasciculus, cingulum, left internal and external capsule | -17, 32, -9 | 0.041 | 5.37 (1.95) |

*Note.* All significant clusters p ≤ 0.05, corrected for multiple comparisons with a cluster size of at least 30 voxels.

The peak coordinates refer to the maximum intensity voxel in MNI 152 standard space. The *p* value is corrected with the threshold-free cluster enhancement.

**Supplementary Table 23.** Significant clusters of associations between MoCA scores and MD at 5-year follow-up with sex and age as covariates.

| **Cluster** | **Number of voxels** | **Anatomical regions** | **Peak coordinates (X, Y, Z)** | ***p* value** | **Peak (mean) t-value** |
| --- | --- | --- | --- | --- | --- |
| 25 | 4729 | Right anterior and posterior thalamic radiation, right anterior corona radiata, right superior longitudinal and inferior fasciculus, right inferior fronto-occipital fasciculus, right uncinate fasciculus, right cingulum, forceps minor, internal and external capsule | 50, -24, 26 | 0.044 | 5.01 (1.93) |
| 24 | 1713 | Left anterior and posterior thalamic radiation, left superior and inferior longitudinal fasciculus, left inferior fronto-occipital fasciculus, left uncinate, forceps major, internal capsule | -47, -35, -8 | 0.045 | 4.41 (2.17) |
| 23 | 818 | Right anterior and posterior thalamic radiation, right superior and inferior longitudinal fasciculus, right inferior fronto-occipital fasciculus, right cingulum, forceps major | 43, -48, 5 | 0.047 | 4.53 (2.08) |
| 22 | 667 | Left anterior thalamic radiation, left anterior corona radiata, left superior longitudinal fasciculus, left inferior fronto-occipital fasciculus, left uncinate fasciculus, forceps minor | -31, 36, 19 | 0.047 | 4.79 (2.35) |
| 21 | 196 | Left anterior thalamic radiation, left superior longitudinal fasciculus, left inferior fronto-occipital fasciculus, left uncinate fasciculus | -41, 25, 14 | 0.048 | 4.63 (2.35) |
| 20 | 127 | Right anterior thalamic radiation, right posterior corona radiata, right cingulum | 13, -49, 48 | 0.050 | 3.72 (2.14) |
| 19 | 110 | Left anterior corona radiata, left inferior fronto-occipital fasciculus, left uncinate fasciculus, left external capsule | -19, 16, -13 | 0.049 | 4.48 (2.84) |
| 18 | 69 | Right inferior fronto-occipital fasciculus, right cingulum, forceps minor | 15, 39, 34 | 0.049 | 4.07 (2.50) |
| 17 | 60 | Right superior longitudinal fasciculus | 43, -44, 34 | 0.050 | 3.14 (2.08) |
| 16 | 58 | Right cingulum | 13, -58, 29 | 0.050 | 4.24 (2.33) |
| 15 | 56 | Right superior longitudinal fasciculus | 38, -60, 37 | 0.050 | 2.94 (2.12) |
| 14 | 55 | Right superior longitudinal fasciculus, right inferior fronto-occipital fasciculus, right cingulum | 25, -45, 43 | 0.050 | 4.06 (2.50) |
| 13 | 33 | Left anterior thalamic radiation, left inferior fronto-occipital fasciculus, left uncinate fasciculus, forceps minor | -16, 51, -4 | 0.049 | 4.49 (3.27) |

*Note.* All significant clusters p ≤ 0.05, corrected for multiple comparisons with a cluster size of at least 30 voxels.

The peak coordinates refer to the maximum intensity voxel in MNI 152 standard space. The *p* value is corrected with the threshold-free cluster enhancement.

**Supplementary Table 24.** Significant clusters of associations between MoCA scores and AD at 5-year follow-up with sex and age as covariates.

| **Cluster** | **Number of voxels** | **Anatomical regions** | **Peak coordinates (X, Y, Z)** | ***p* value** | **Peak (mean) t-value** |
| --- | --- | --- | --- | --- | --- |
| 21 | 12240 | Body and splenium of corpus callosum, anterior thalamic radiation, left posterior thalamic radiation, left corticospinal tract, left superior and posterior corona radiata, left superior and inferior longitudinal fasciculus, left inferior fronto-occipital fasciculus, left uncinate fasciculus, left cingulum, forceps major, fornix, left internal and external capsule | -49, -15, -19 | 0.022 | 5.24 (1.70) |
| 20 | 6297 | Splenium of corpus callosum, right cerebral peduncle right anterior and posterior thalamic radiation, right posterior corona radiata, right superior and inferior longitudinal fasciculus, right inferior fronto-occipital fasciculus, right uncinate, right cingulum, forceps major and minor, fornix, right internal and external capsule | 35, -55, 15 | 0.029 | 5.13 (1.76) |
| 19 | 4051 | Left anterior thalamic radiation, left anterior corona radiata, left superior longitudinal fasciculus, left inferior fronto-occipital fasciculus, left uncinate, left cingulum, forceps minor, left external capsule | -19, 16, -13 | 0.033 | 6.36 (1.80) |
| 18 | 2187 | Right anterior thalamic radiation, right anterior corona radiata, right superior longitudinal fasciculus, right inferior fronto-occipital fasciculus, right uncinate fasciculus, right cingulum, forceps minor | 31, 14, 43 | 0.040 | 4.35 (1.94) |
| 17 | 762 | Right anterior thalamic radiation, right posterior corona radiata, right superior and inferior longitudinal fasciculus, right inferior fronto-occipital fasciculus, right cingulum, forceps major | 25, -45, 45 | 0.045 | 4.77 (1.75) |
| 16 | 579 | Right superior longitudinal fasciculus | 47, -24, 30 | 0.043 | 4.55 (1.89) |
| 15 | 178 | Right anterior thalamic radiation, right internal capsule | 16, 0, 8 | 0.047 | 3.50 (1.93) |
| 14 | 142 | Right corticospinal tract | 13, -26, 65 | 0.049 | 4.07 (1.79) |
| 13 | 102 | Right anterior thalamic radiation | 42, 29, 27 | 0.049 | 3.51 (2.05) |
| 12 | 95 | Right superior longitudinal fasciculus | 10, -48, 51 | 0.049 | 2.92 (1.77) |
| 11 | 80 | Right inferior fronto-occipital fasciculus, right cingulum, forceps minor | 16, 38, 33 | 0.048 | 4.18 (2.14) |
| 10 | 65 | Right superior and posterior corona radiata, right corticospinal tract | 20, -33, 38 | 0.048 | 3.64 (2.08) |
| 9 | 46 | Right anterior thalamic radiation, right corticospinal tract right internal capsule | 18, -7, 6 | 0.049 | 3.01 (1.84) |
| 8 | 36 | Right superior and inferior longitudinal fasciculus | 56, -24, -13 | 0.049 | 3.12 (2.35) |

*Note.* All significant clusters p ≤ 0.05, corrected for multiple comparisons with a cluster size of at least 30 voxels.

The peak coordinates refer to the maximum intensity voxel in MNI 152 standard space. The *p* value is corrected with the threshold-free cluster enhancement.
